# One-step visualization of natural cell activities in non-labeled living spheroids

**DOI:** 10.1038/s41598-022-05347-z

**Published:** 2022-01-27

**Authors:** Shotaro Tanaka, Kotaro Takizawa, Fumio Nakamura

**Affiliations:** Department of Biochemistry, Tokyo Women’s Medical University School of Medicine, 8-1 Kawada-cho, Shinjuku-ku, Tokyo, 162-8666 Japan

**Keywords:** Cellular imaging, Drug screening

## Abstract

3D cultured cell aggregates, including spheroids, reflect the gene expression patterns of living tissues/organs. Mass preparation of spheroids enables high-throughput drug screening (HTS). However, conventional optical imaging of spheroids makes it difficult to obtain sufficient resolution of individual living cells in the thick cellular stack. Rapid and accurate assessment of cellular responses in spheroids is required for effective drug screening. Here, we show that negative contrast imaging (NCI) of spheroids overcomes this issue. Hydrophilic fluorescent dye added into the culture medium rapidly diffused into the intercellular space of living spheroids within a few minutes. Confocal microscopy showed the NCI of individual cells as dark and detailed contours clearly separated with fluorescence signals in the intercellular space. NCI enables the visualization of the alteration of cell morphology after anti-tumor drug application to living spheroids and the measurement of the fluorescent dye diffusion rate without any complicated pretreatments. Using this system, we found that the antitumor drug doxorubicin reduced the intercellular space of spheroids consisting of the human hepatocyte carcinoma cell line HepG2, through the activation of TGF-β signaling and upregulation of ECM protein expression, implicating a drug resistance mechanism. Collectively, the combination of NCI of spheroids and HTS may enhance the efficiency of drug discovery.

## Introduction

Rapid and effective drug development is a constant social need that prompts researchers and the drug discovery industry to adopt more appropriate preclinical experimental models that reflect actual tissue characteristics^1,2^. Compared to conventional monolayer cell culture models, self-assembled cell aggregates, including spheroids and organoids, possess more similar gene expression patterns and phenotypes to in vivo tissue^1,2^. Therefore, cell aggregates are being increasingly utilized for drug discovery and cell toxicity studies^3–7^.

Spheroids can be quickly prepared from cancer cell lines as relatively uniform cell aggregates. They have been utilized in high-throughput screening (HTS) for drug discovery^4^. As the spheroid core retains a nutrient and O_2_-starved environment similar to the tumor region distant from blood vessels, the core cells are thought to acquire a chemotherapeutic resistant phenotype^5^. Therefore, observing the drug response of the spheroid core region is the most important evaluation in screening antitumor drug candidates using spheroids.

However, present evaluation methods that almost utilize fluorescent microscopy with specific fluorescence-labeled target molecule are unable to collect details of various cellular drug responses. When optical microscopic acquisition is undertaken, light scattering, low light penetration, and auto-fluorescence interfere with high-resolution images of the cells inside the cell aggregates, even if the target is specifically labeled with a fluorophore^6^. To date, the only way to evaluate the drug effect on living spheroids is to observe the growth of the whole structure of the cell aggregates^4,8^ and the staining of live/dead cells^7^. Immunostaining of fixed spheroids is therefore a useful way to evaluate drug effects in the core region of the cell aggregates at the molecular level. However, such a method may require many samples and is a time-consuming process. To accelerate drug screening, periodic observation and multi-item inspection of cell aggregates are necessary.

Here, we describe an application of NCI on live spheroid for the first time. NCI showed great results on the study for organelle^9^ or neuronal structure in living brain previously^10^, because of its unique features such as visualizing non-fluorescence labelled structures. We are firstly reporting its advantages that overcomes the above-mentioned issues and enables one to undertake timed and multi-item observations, including structural changes and dynamics of the core cells at the single-cell level. The method is a simple, one-step treatment, and is easy to integrate with confocal microscopy; thus, it can be utilized in conventional HTS, as well as for basic research.

## Result

### Evaluation of NCI for instant observation

**Figure 1:**
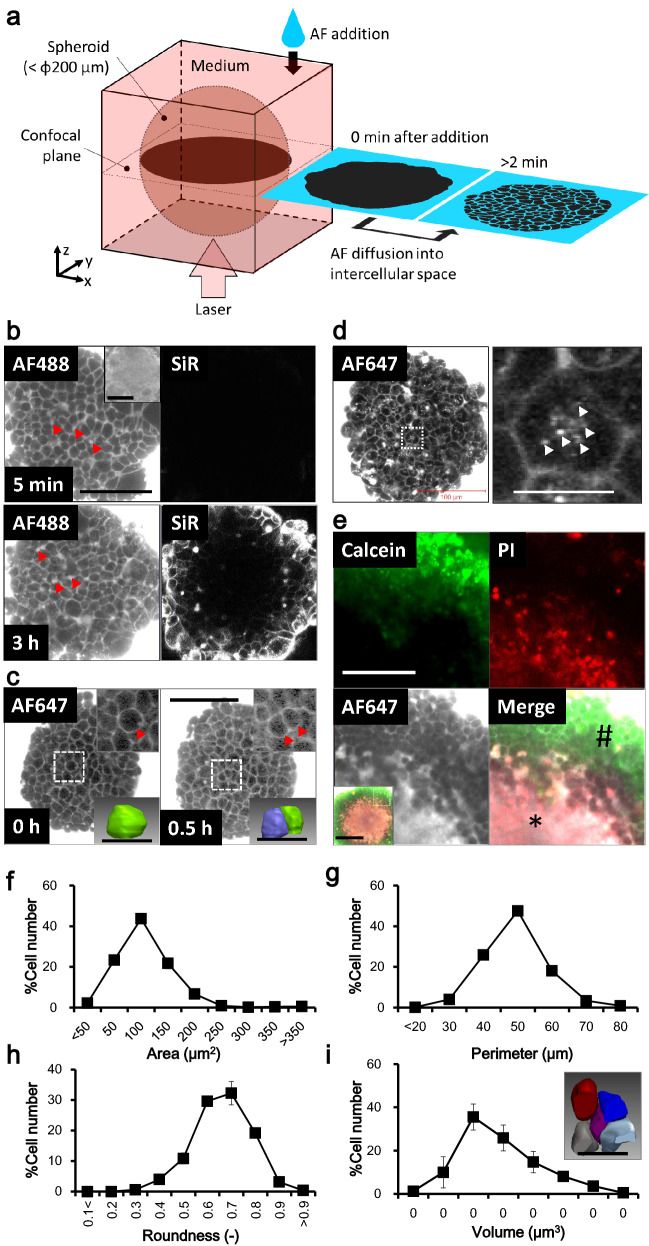
NCI visualizes the structure and activity of individual living cells in the spheroid core. (**a**) Schematic imaging principle of spheroid NCI. Diffusion of the AlexaFluor (AF) fluorescent dye into the spheroid is observed by confocal microscopy. Individual living cells in the spheroid are visualized as a dark shadow of the cell contour. (**b**) Comparison of the two confocal images of the same HepG2 spheroid visualized by NCI (AF488) and by the representative conventional living cell staining method, SiR-actin (SiR), at 5 min and 3 h after dye addition into the medium. Bar: 100 µm. Arrowheads: bile canaliculus-like structures. Inset: Bright field. Bar: 100 µm. (**c**) Visualization of cytokinesis of a single representative HepG2 spheroid cell unfolding over time. The spheroid incubated at 37 °C and 5% CO_2_ in a humidified incubator on the stage of the microscope was scanned at 0.5-h intervals. The AF647 was added once at the first NCI acquisition. Bar: 100 µm. Upper inset: enlarged image of the dashed rectangle. Arrowhead: the parent- and daughter cell(s). Lower inset: reconstructed 3D structure of the target cell(s). Bar: 20 µm. (**d**) Visualization of constitutive endocytosis of the HepG2 spheroid cells after 1 h incubation with AF647-containing medium. Left: NCI of the whole spheroid. Bar: 100 µm. Right: enlarged image of the dashed rectangle. Arrowheads: fluorescent punctate dots in the representative cell. Bar: 20 µm. (**e**) Visualization of living/dead cells by NCI with 20 × objective lens. Day 16 HepG2 spheroid was observed by multicolor fluorescent confocal microscopy. Living cells (calcein positive: green, sharp) and dead cells (PI positive: red, asterisk) were clearly identified by NCI (AF647, FR) by fluorescent accumulation. Bar: 50 µm. Inset: whole image with merge. Dashed rectangle: the enlarged area in (**e**). Bar: 100 µm. (**f**–**h**) Histograms of the individual cell contours in the confocal image of the HepG2 spheroid analyzed by the (**f**) the area, (**g**) the perimeter, and (**h**) the roundness. N = 6. (**i**) Histograms of the individual cell volumes in HepG2 day 5 spheroids. N = 4. Inset: re-constructed 3D structures of the represent cells. Bar: 20 µm.

NCI observes a negative contrast image of a target of interest. The target structure in fluorescent dye-filled space excludes the surrounding fluorescent dye by its volume; thus, confocal imaging visualizes the weak (or no) fluorescence area, such as the target’s shadow (Fig. 1a). NCI has been utilized for organelle visualization^9^ and the brain^10^ without any specific labeling of the target.

We hypothesized that the addition of a hydrophilic fluorescent dye into the medium could aid the visualization of the living cells in the spheroid by NCI. We added a commercially available hydrophilic fluorescent dye, Alexa Fluor (AF) 488-NHS (MW, 643.4 Da), to the medium in which HepG2 spheroids were cultured. The dye immediately diffused into the intercellular space of the living spheroid cells and became visible within two minutes, but not in the cytoplasm of cells. The individual living cell bodies with weak fluorescent signal in the intercellular space were outlined in detail by confocal microscopy (Fig. 1b left). While other hydrophilic fluorescent dyes including AF647 (MW, 1025.2 Da) also gave similar confocal images (Fig. 1c,d), hydrophobic dyes showed predominant accumulation in the peripheral cells of the spheroids (Extended Data Fig. 1, Rhodamine 6G (Rh6G) and^11^). We then examined the NCI of spheroids with HEK293T cells expressing membrane-anchored AcGFP. The AcGFP signal and the external AF647 signal were well aligned (Extended Data Fig. 2). This indicated that the diffused AF647 signal clearly marked the extracellular space of the spheroid. Furthermore, AF488-conjugated albumin (MW, 66.5 kDa) took a longer time to diffuse into the intercellular space (Extended Data Fig. 3). We concluded that small (~ 1000 Da of MW) hydrophilic dyes are required for NCI of spheroids. Under these conditions, we could identify the individual spheroid cells located at a depth of 100 μm from the adhesion plane (Extended Data Fig. 4).

Next, we compared the performance of NCI with that of conventional imaging methods for living cells. One membrane-permeable fluorescent dye, SiR-act, can stain the cytoskeleton of living monolayered cells^12^. We used multicolor confocal microscopy of SiR-act as far-red fluorescence and AF488 for NCI as green fluorescence on a single HepG2 spheroid. AF488 signals diffused into the spheroid core within 5 min with clear visibility of individual cellular structure (Fig. 1b, upper left). In contrast, SiR-act required several hours to show clear fluorescent signals, with little fluorescence at the core region (Fig. 1b, lower right). These data showed that NCI significantly improved the performance of imaging, offering a rapid and deep field of view for individual spheroid cells, compared to conventional live cell imaging methods.

### Evaluation of NCI for long-term observation

NCI can also enable the long-term observation of living spheroids. Time course observation of single spheroids at 30 min intervals with a single AF647 addition at the starting point showed cytokinesis of the cells in the spheroid core. The spherical parent cell (Fig. 1c, left, dashed rectangle, and inset) was divided into two daughter cells (Fig. 1c, right, dashed rectangle, and inset). Sequential confocal tomograms gave the 3D structure and cell volume, and the daughter cells (Fig. 1c right, lower inset, 1066 and 1165 μm^3^) were accurately measured to be one-half the volume of their parent cell (Fig. 1c left, lower inset, 2211 μm^3^). An hour after dye addition, we also observed punctate small fluorescent spots within the spheroid core cells, indicating endocytosis (Fig. 1d right, arrowhead). Spheroids cultured for long periods expand in size, and the core cells begin necrosing due to poor nutrition and oxygen^5^. The NCI of day 16 HepG2 spheroids showed fluorescence accumulation in the core region that partially merged with signal from the cellular necrosis indicator propidium iodide (PI) (Fig. 1e, asterisk), and clear separation of individual cells in the peripheral region, stained with the living cell indicator calcein, was evident (Fig. 1e, sharp). This indicates that hydrophilic dye accumulation in spheroids can also be used as a cell death indicator in NCI.

Interestingly, NCI also visualized contaminated noncellular components that can clearly be distinguished from spheroid cells. Such components often existed in the medium, and likely come from the serum and/or culture device; sometimes the cells assemble on the debris (Extended Data Fig. 5, arrowhead). This suggested that NCI may be suitable for studying the interaction of cellular-noncellular components (such as bone, cartilage, and artificial materials).

### Measurement of inflow diffusion rate of AF by an NCI-spheroid model

**Figure 2:**
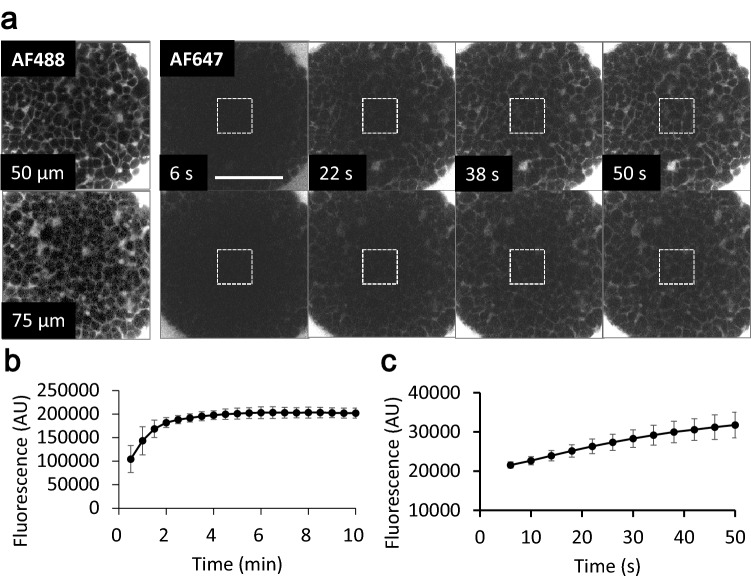
Measurement of the inflow diffusion rate of AF647 in HepG2 spheroid cores. (**a**) A single spheroid was visualized with NCI by addition of AF488 as an indicator to set the position of confocal microscopy scanning (left). Then AF647 was added into the medium, and scanned at distances of 0, 25, 50 and 75 µm from the adhesion plane at 4 s intervals for 50 s. The gain in fluorescent intensity at the deeper spheroid core (75 µm) was lower than that of the shallow core (50 µm). Dashed rectangles indicate the area for quantity fluorescent intensity. Bar: 100 µm. (**b**,**c**) Graph of changing the fluorescent intensity over long (**b**) and short (**c**) periods at a distance of (**b**) 70 µm or (**c**) 75 µm from the adhesion plane of the target spheroid. N = 3 (**b**) or 5 (**c**). Within 5 min, the fluorescence of the core reached a plateau.

The diffusion rate of drugs of interest into living tissues is an important factor for drug effectiveness. Several factors affect the diffusion of drugs, including ECM structure and density, cell adhesion, and the cell body itself which control the width of the intercellular space. Cell aggregates are regarded as a potential model to measure the drug diffusion rate in tissue. We measured the diffusion rate of AF647 using NCI. Before the addition of AF647 into the spheroid medium, we undertook NCI, using AF488, and manipulated the scanning area and the distance from the adhesion plane (0, 25, 50, and 75 μm) (Fig. 2a, left column). To observe saturation of dye diffusion, 30 s after addition of AF647 into the medium, confocal images were acquired at 30 s intervals for 10 min. The intensity gain of AF647 fluorescence at the spheroid core was saturated within 4 min (Fig. 2b). To calculate the diffusion rate, time course image acquisition in short periodic intervals was performed (Fig. 2a right, c). The diffusion rate from the linear approximation curve created within 6 to 26 s was 293.98 s^−1^. These data demonstrated the ability of NCI to measure the diffusion rate of various compounds into the spheroid, although even if we have to do fluorescent labeling. Combined with the endocytosis observations (Fig. 1d), this method can measure the diffusion rate of drug transport from the outside into the cytoplasm of the spheroid cells.

### Distinct NCI patterns for spheroids of different cell lines

**Figure 3:**
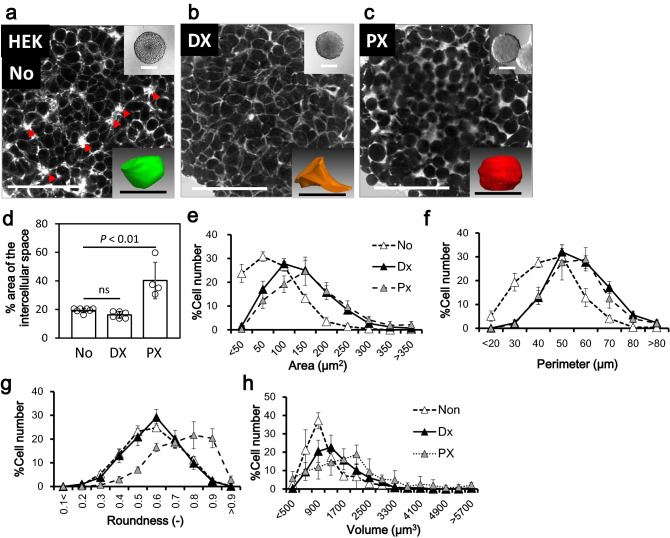
Structural change of HEK293T spheroid cells induced by antitumor drug treatment. (**a**–**c**) Representative NCI image of HEK293T day 5 spheroids without (**a**) or with overnight (18 h) treatment of doxorubicin (DX) (**b**) or paclitaxel (PX) (**c**). The drugs can be seen to make the individual cell structure and spheroid packaging either tight (**b**) or loose (**c**). Arrowheads: The characteristic Rosetta structure. Bar: 50 µm. Inset upper: Bright field. Bar: 200 µm. Inset lower: a reconstructed 3D structure of the represent cell. Bar: 10 µm. (**d**) The ratio of intercellular area to the total area of one confocal image of the spheroid. N = 7 (No); 6 (DX), 4 (PX). (**e**–**g**) Histograms of the individual cellular contours in the confocal image of the spheroid analyzed with the (**e**) area, (**f**) perimeter, and (**g**) roundness. N = 6. (**h**) Histograms of the volume of the individual spheroid cells located in the 50 × 50 × 100 µm of virtual area set in the center of the scanned spheroid. N = 3.

Spheroids are now regarded as a standard model for HTS. We evaluated whether the NCI of spheroids was suitable for HTS imaging. We first examined whether NCI could reveal distinct characteristics of different cell lines, including HEK293T, HepG2, and the human colon cancer-derived cell line HCT116. We found clear differences in the cellular filling patterns of these cell lines. The filling pattern of HepG2 spheroids showed a compacted appearance resembling paving stones together with bile canaliculus-like structures (Fig. 1b, arrowhead^13,14^), compared with the relatively sparse cellular filling of the HEK293T spheroid (Fig. 3a left). The HEK293T spheroids showed Rosetta structures within the core (Fig. 3a, arrowhead), which likely represent vigorous cell proliferation. Interestingly, the HCT116 spheroids showed a biphasic structure with a compressed core and sparse periphery (Extended Data Fig. 6a, dashed line indicates the border). The extracted contours of the individual cells from the spheroid confocal images provided a quantitative evaluation of the characteristics of the three spheroids in cross-sectional area, perimeter, and roundness (Figs. 1f–h, 3e–g triangle, Extended Data Fig. 6b–d). Connecting of the cellular contours extracted from the sequentially acquired confocal images of the spheroid also provided 3D cell structures, together with volume histograms and characteristic shapes (Figs. 1i, 3a inserted and h triangle, and Extended Data Fig. 6e,f). These data indicated that NCI can provide clear and varied information about changes in cellular structural phenotypes, such as cell differentiation.

We next examined whether NCI can identify the effects of culture conditions and culture times on spheroid structure and viability. We compared two culture conditions, the low-adhesion plate used in the present study, and the hanging drop method. After 5 days of incubation, the spheroids under these two conditions showed little difference in appearance, viability, and individual cellular volume (Extended Data Fig. 7a). In contrast, after 12 days of incubation, the spheroids from the low-adhesion plate showed marked cell death compared with the hanging-drop method (Extended Data Fig. 7b–d). Additionally, the histogram of the cellular volume was clearly less in the low-adhesion plate spheroids, than in the hanging drops (Extended Data Fig. 7e). Enlargement of spheroids may cause insufficient medium diffusion, hypoxia, and low nutrition in the core region, causing both cell differentiation and cell death. Thus, NCI can show various characteristics of spheroid cells, including cell death and differentiation, supporting its use for HTS.

### Alteration of spheroidal structure with anti-tumor drugs

Next, we attempted to observe the drug response of spheroids by NCI. HEK293T spheroids were treated overnight (18 h) with the two antitumor drugs, the multifunctional antitumor drug doxorubicin (DX) and microtubule stabilizer paclitaxel (PX). Both of the drugs conferred only slight changes in the whole spheroid size observed by conventional optical imaging (Fig. 3a–c, insert), but there was a clear difference in the cellular filling structure by NCI. DX treatment eliminated the Rosetta structure observed in non-treated spheroids (Fig. 3a, arrowhead), and induced tight cellular fillings (Fig. 3b). This may be due to the volume increase of the cells from DX, which arrests cells in G2 phase. In contrast, PX treatment induced expansion of the intercellular space together with a morphological change of the cells to a spherical shape (Fig. 3c), probably due to the inhibition of microtubule turnover. These changes were also apparent in a quantitative fashion in terms of a change in the intercellular space (Fig. 3d), the individual cell contour (Fig. 3e–g) and the 3D structure (Fig. 3h). To our knowledge, this is the first report of such drug-induced structural changes in spheroid cells.

### Doxorubicin-induced ECM expression in spheroids of HepG2 cells

**Figure 4:**
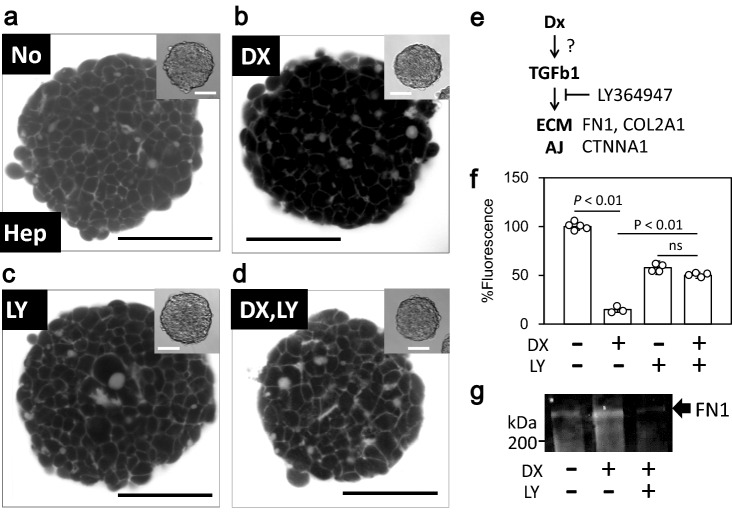
Doxorubicin reduces fluorescent dye diffusion into the HepG2 spheroid core region by the TGF-β–ECM signaling cascade. (**a**–**d**) Representative confocal image of living HepG2 spheroids treated with DX, LY (LY364947, TGF-β inhibitor), or both. (**b**) Shows a darker core than (**a**); (**c**,**d**) contain higher intensity of fluorescence than (**b**). Bar: 100 µm. Inset: bright field. Bar: 50 µm. (**e**) Simplified schema of TGF-β signaling cascade with related genes. TGFB1: TGF-β 1, FN1: fibronectin 1, COL2A1: collagen 2A1, CTNNA1: catenin A1, AJ, adherent junctions. (**f**) Quantitative analysis of the fluorescent intensity in confocal images of the target spheroid in (**a**–**d**). N = 5 (DX−/LY−); 4 (DX−/LY+); 3 (DX+/LY−); 4 (DX+/LY+). (**g**) Immunoblotting for fibronectin on the drug-treated spheroids. DX induces FN1 expression and LY suppresses the effect. The entire blot with membrane edges visible is shown in Extended Data Figure S10.

The DX-treated HepG2 spheroids showed a more intriguing phenomenon. Non-treated HepG2 spheroids showed clear and compacted intercellular spaces in the core region (Figs. 1b and 4a), indicating smooth diffusion of the dye into the space. However, DX treatment reduced the fluorescence intensity in the core region, and produced a less clear intercellular space (Fig. 4b). To uncover the concerning genes, we undertook RNAseq analyzing against the 4 experimental group designated by culture style (monolayer culture or spheroid) and by with/without DX treatment. The transcriptomic analysis of the DX-treated spheroids showed clear difference of gene expression pattern among the experiment group (Extended Data Fig. 8c, S9, S10) and the activity of several gene function pathways including the TGF-β signal cascade (Extended Data Fig. 8d, asterisk), as well as the upregulation of some ECM components, including collagens and fibronectin (Table 1 and Extended Data Fig. 11). Although such the upregulation of the genes were not specific on spheroid, we simultaneously applied DX and the TGF receptor specific inhibitor LY36497 to determine whether the TGF-β signaling cascade is associated with the decrease in fluorescence intensity. As expected, co-administration of LY36497 reduced the effect of DX (Fig. 4d,f), and also suppressed DX-induced fibronectin expression (Fig. 4g and Extended Data Figs. S12, S13). These results suggest that DX may promote the expression of ECM proteins through the TGF-β signaling cascade to reduce the AF diffusion efficacy by upregulating ECM density.

**Table 1:** Differentially expressed genes (fpkm).

Gene	Non_Sphe	Dx_Sphe	Non_Mono	Dx_Mono
TGFB1	61	87	121	353
COL21A1	78	116	97	68
COL2A1	171	312	157	288
FN1	1416	2009	2961	1844
LAMA5	184	268	495	655
LAMC1	151	200	151	162
ITGB1	328	481	389	378
CDH1	44	50	20	37
CTNNA1	355	443	286	327

*Sphe* spheroid, *Mono* monolayer culture.

## Discussion

In the present study, we demonstrated the utility of NCI in living spheroid cell imaging. Simple addition of the hydrophilic fluorescent dye into the medium provided quite dramatic results, within a few minutes, for the confocal image, with well-separated individual cell bodies in the core region of the spheroids. Image analysis provided quantitative results, such as individual cell volume. This minimally invasive imaging method revealed antitumor drug-induced changes in cell structure and the molecular diffusion of drug into the intercellular space, which can be used to immediately evaluate drug effects.

We demonstrated that NCI can detect detailed shapes of individual cells of the spheroid core without invasive manipulation, which represents a breakthrough overcoming technical limitations of more conventional methods. By observing spheroids with NCI, we were able to visualize drug responses of the spheroids in terms of structural (Fig. 3) and dye-diffusion characteristics (Fig. 4), differences in cell lines (Fig. 1, 3, and Extended Fig. 6), cellular viability (Fig. 1 e), cellular functions such as cytokinesis (Fig. 1c) and endocytosis (Fig. 1d) and non-cellular components (Extended Fig. 5). This was all possible with the simple addition of hydrophilic fluorescent dye to the medium for just a few minutes. These advantages were mainly due to the rapid inflow diffusions of the dyes we used (Fig. 2), compared with dyes used in previous studies^15–17^. The significant expansion in parameters that one can visualize, as well as the shortening of the observation time and the simplification of the process, should facilitate studies of drugs of interest using this model. At the same time, single cell-level observation can minimize the impact of spheroid variation, and thereby facilitate quality control. Of note, these other methods can only utilize single wavelengths, whereas in the present study, we utilized a total of three wavelengths for fluorescent imaging (Fig. 1e) as well as a bright field image. This suggests that NCI can improve confocal microscopy, and simultaneously facilitate both HTS and basic research. In combination with the molecular imaging of specific genes, NCI can provide structural data at the single-cell level, visualize the response of individual cells to drugs of interest, and enhance the progress of drug discovery. Further, non-cellular components can be observed by NCI (Extended Fig. 5), but not by conventional living cell imaging methods. The ability to identify non-cellular components for checking and controlling the quality of spheroids should also be helpful for drug discovery. NCI may also be suitable for the study of cellular/noncellular components, such as bone, cartilage, and artificial materials, and may be important for the field of regenerative medicine in the future.

We also demonstrated that NCI can be useful for the evaluation of the response of the spheroid to certain drugs, with a readout of an alteration of dye diffusion into the spheroid core. We have highlighted the effect of DX on the hepatoma cell line spheroids (Fig. 4). DX has multiple clinical effects such as apoptosis induction by accumulation in mitochondria, ROS generation and inhibition of transcription^18^, and we surely detected upregulation of ribosomal proteins (Extended Data Figs. 9, 10, asterisk). That is therefore regarded as a first choice for treatment of some tumors^19^. However, longitudinal administration of DX often causes fibrosis in healthy tissues, including the liver^20^ and cardiovascular endothelium^21^. Furthermore, tumor fibrosis caused by antitumor drugs, including DX, is now a serious concern because it reduces the transport efficiency of the drug into the affected area^22^, thus demanding the simultaneous application of anti-fibrosis drugs^23^. Using NCI, we demonstrated a correlation between DX stress on spheroid cells and the reduction of the dye diffusion rate into the spheroid core due to increased expression of ECM proteins. It has also been suggested that ECM upregulation is triggered by the TGF-β signaling cascade^24^. We confirmed this by using a specific inhibitor of the TGF-β receptor and observing recovery of the dye diffusion rate into the spheroid core (Fig. 4). Of note, the result indicated that the NCI-based spheroid assay could be utilized both for evaluation of drug-induced ECM augmentation causing tissue fibrosis, and as a screening assay for drug candidates that suppress fibrosis. Overall, our results demonstrated the utility of NCI for HTS drug screening.

There remain two tasks for the actual application of NCI to HTS. The first is the difference in the degree of cell packaging among the cell types that affects dye diffusion into the spheroid core. As shown by the HCT116 spheroid in Extended Data Fig. 6, tightly packaged spheroids had lower dye diffusion rates and less intense fluorescence signals in the intercellular space. The affinity of cell–cell adhesions, such as in tight junction formation, may affect the dye diffusion rate. Together with the ability to observe deep inside the spheroid, as shown in Extended Data Fig. 4c, the image sharpening and ability to detect weak fluorescent signals may overcome this issue. The other task is to automate image processing and analysis. The NCI of spheroids provides a significant amount of information in a single spheroid study. To ensure reproducibility and shorten analytical time, automatic analysis should be performed, especially to translate drug-induced structural changes to the study of drug mechanisms.

In conclusion, we have developed a simple and effective live imaging method, NCI, which can measure many parameters of individual cells in the spheroid core region. Simple and rapid visualization with low invasiveness should be suitable for studies utilizing primary cultured cells and stem cells that are difficult to manipulate genetically. NCI also uncovered the cell line-dependent cell packing pattern and unique structural changes in response to anti-tumor drugs. Transcriptomic analysis of conditioned spheroids provided gene candidates corresponding to tumor chemoresistance or other treatment measures. NCI of 3D cultures of tumor cells could therefore contribute substantially to studies on anti-cancer drug development and chemoresistance.

## Methods

### Reagents, antibodies, and plasmids

AF488 N-hydroxysuccinimide (NHS) ester and AF647 NHS ester were purchased from Thermo Fisher Scientific (Waltham, MA, USA). Rhodamine 6G (Rh6G) was obtained from Sigma-Aldrich (St. Louis, MO, USA). Calcein AM was obtained from Dojindo (Osaka, Japan). SiR-Actin and the calcium channel inhibitor verapamil were obtained from Cytoskeleton (Denver, CO). The TGF-β receptor I inhibitor LY364947, PI, DX, and phenylmethylsulfonyl fluoride (PMSF) were obtained from Fujifilm (Tokyo, Japan). Rabbit polyclonal anti-fibronectin 1 antibody was purchased from Abcam (ab2413) (Cambridge, UK). pAcGFP1-Mem was purchased from Clontech (Takara Bio, Shiga, Japan). Smear Gell (product number: SG-01) for spheroid immunofluorescence was obtained from GenoStaff (Tokyo, Japan). The SlowFade mountant as a microscope was obtained from Thermo Fisher Scientific. The protease inhibitor cocktail (P8340) was purchased from Sigma-Aldrich.

### Cell culture

The HepG2 human hepatoma cell line (JCRB1054) and the HCT-116 human colon cancer cell line (JCRB1408, stably expressing luciferase) were obtained from the Japanese Collection of Research Bioresources Cell Bank (Osaka, Japan) and tested for mycoplasma contamination. The HEK293T human embryonic kidney cell line was described previously^25^. Cells were cultured at 37 °C and 5% CO_2_ in a humidified incubator in RPMI-1640 supplemented with l-glutamine and phenol red supplemented with 10% fetal bovine serum (Thermo Fisher Scientific). HEK293T cells stably expressing plasma membrane-anchoring AcGFP were prepared with pAcGFP1-Mem transfection and selection using G418 resistance. DX treatment (final concentration: 0.5 µg/mL (0.86 µM)) on the spheroid was continued for 18 h in a humidified incubator under the same conditions. PX (final concentration: 1.0 µM) and LY364947 (final concentration: 0.4 µM) were used for 18 h.

### Spheroid preparation

Cultured cells grown in a 10 cm diameter plastic culture dish for spheroid preparation were trypsinized and suspended in phosphate-buffered saline (PBS). The cell number was counted using a Coulter cell counter (Scepter, Merck Millipore, MA, USA) and transferred to 96-well U-bottomed low-adhesion plates (Prime Surface 96U, SUMITOMO BAKELITE, Tokyo, Japan) with 80 cells per well in 100 µL of the corresponding medium. After 5 days of incubation in a humidified incubator at 37 °C and 5% CO_2_, cells formed spherical shapes with diameters of approximately 200 µm (HepG2) or 300 µm (HEK293T and HCT-116). Before microscopic observation, the spheroids were collected by pipetting them individually with a 1000 µL tip and placed in a 35 mm diameter glass-bottomed dish (Matsunami, Osaka, Japan) or Nunc Lab-Tek Chambered Coverglass (Thermo Scientific) with 300 µL of the corresponding medium. For the hanging drop method, we utilized GravityPLUS (InSphero, Schlieren, Switzerland) according to the manufacturer’s instructions.

### Western blotting

Unless otherwise stated, the following process was performed at room temperature (25–30 °C). A total of 40 targeted spheroids were collected into one 1.5 mL low-adhesion microtubes using a pipette. The medium was carefully removed, and spheroids were washed twice with Tyrode’s buffer containing phenylmethylsulfonyl fluoride (Fujifilm) and protease inhibitor cocktail (Sigma). The pellet was suspended in 40 µL of 1 × sample buffer, sonicated for 15 min at 4 °C, and incubated at 100 °C for 5 min. A 15 µL sample of the supernatant was subjected to SDS polyacrylamide gel electrophoresis (PAGE, 8% acrylamide gel, 75 min at 30 mA constant current), and the gel was electroblotted onto a Immobilon FL membrane (Millipore) for 90 min at 13 V. The membrane was then blocked in Tris-buffered saline (TBS) buffer with 1% (w/v) skim milk at 4 °C overnight with gentle shaking. The membrane was then incubated in 1% (w/v) skim milk–0.1% Tween20-containing TBS buffer with anti-FN1 antibody (final concentration: 2 µg/mL) and incubated for 1 h. The membrane was washed and suspended in 1% (w/v) skim milk–0.1% Tween20–0.1% SDS-containing TBS buffer with IRDye800-labeled anti-rat Ig antibody solution (final concentration: 0.1%) for 45 min. After three washes with 0.1% Tween20-containing TBS buffer and once with TBS buffer, the membrane was dried, and the fluorescence signals were scanned using an Odyssey system (LI-COR, Lincoln, NB, USA).

### Immunofluorescence

Five targeted spheroids were pipetted into one 1.5 mL low-adhesion microtube. The pellet was washed once with PBS containing PMSF (1 mM) and protease inhibitor cocktail (Sigma). The pellet was suspended in 3 µL of the same PBS and placed on a 35 mm glass-bottomed dish (Matsunami). The pellet was enclosed in a Smear Gell, according to the manufacturer’s instructions. Then, the polymerized gel attached to the glass-bottomed dish was fixed by adding 200 µL of 4% PFA-PBS for 10 min. The solution was aspirated and dialyzed with 3% (w/v) BSA–1% Tween20 containing PBS for 2 h. The gel was incubated with 200 µL of the same solution containing rat anti-FN1 antibody (10 µg/mL) with gentle rocking and shaking overnight at 4 °C. The gel was washed three times and incubated in 0.9 mL of the same solution containing 2.6 µg of AF-labeled donkey anti-rabbit Ig for 2 h. The gel was washed three times and treated with SlowFade (Thermo Fisher Scientific) for protection against photo-bleaching.

### Confocal microscopy

Unless otherwise specified, the following processes were performed at room temperature: AF NHS ester and other activated fluorescent dyes were diluted in dimethyl sulfoxide (DMSO) at 10 mg/mL and stored at − 20 °C. To neutralize the active group, 10 µL of the stock solution was incubated in a 1.5 mL microtube with 500 µL of RPMI-1640 containing 10% FCS for 1 h with gentle rotation in the dark. After neutralization, the solution was centrifuged at 20,000×*g* at 4 °C for 5 min, and the supernatant was collected. A 30 µL sample of the supernatant was added to 300 µL of spheroid-containing medium by gently pipetting several times. The dish was then incubated in a humidified incubator for the required time at 37 °C and 5% CO_2_ until microscopic observation. To compare the living cell imaging ability of NCI and SiR-actin staining, SiR-actin (final concentration: 3.3 µM), verapamil (10 µM), and 30 µL of AF488 were simultaneously added to 300 µL of the medium.

The prepared spheroids in the glass-bottomed dish were imaged on a Zeiss LSM510 or LSM710 confocal microscope with a C-Apochromat 40 × /1.2 W water immersion lens and ZEN software (Zeiss, Jena, Germany). The cells were visualized without changing the medium to avoid stress or morphological changes in the cells. Confocal images were acquired at a distance of 50 µm from the adhesion plane of the spheroid. Two images were acquired for averaging at a pixel size of 512 × 512 (scaling: 0.415 × 0.415 µm) with 1.0 µm pinhole diameter as the software-recommended scan speed. AF488, calcein, and AcGFP were excited at 488 nm using an argon laser at a power of 2.0%. Red fluorescent dyes, PI and Rh6G, were excited at 543 nm using a helium/neon laser at 2.0% power. AF647 and SiR-actin were excited at 633 nm using a helium/neon laser at 2.0% power. Serial confocal images for z stacking were acquired at 1.0 µm intervals starting at the adhesion plane of 0 µm.

For cell viability imaging shown in Fig. 1e, calcein (final concentration: 3.3 µM) and PI (1.6 µg/mL) were added to the medium and incubated 18 h prior to imaging, after which 30 µL of AF488 was added before imaging. The prepared spheroids in the glass-bottomed dish were imaged on a Zeiss LSM710 confocal microscope with EC Plan-Neofluar 20 × /0.50 M27 correction and ZEN software (Zeiss). Confocal images were acquired at a distance of 75 µm from the adhesion plane of the spheroid. Sixteen images were acquired for averaging at a pixel size of 1024 × 1024 (scaling: 0.415 × 0.415 µm).

### Measurement of AF diffusion rate

Before measurement, the prepared spheroids were transferred into 8 well LabTek chambered cover glasses with 300 µL of the medium and incubated for 3 h at 37 °C and 5% CO_2_ for adhesion of the spheroid on the chamber. The chamber was then set on the microscope stage on an LSM710 confocal microscope with a C-Apochromat 40 × /1.2 W correction and ZEN software (Zeiss). First, for the z position setting, 20 µL of AF488 stock solution was mixed into the chamber. After 5 min, the z-position of the spheroid was adjusted by NCI excitation at 488 nm. Then, 100 µL of the medium was removed from the chamber and 30 µL of AF647 stock solution was mixed and returned to the chamber at the start of the measurement. For the long periodic observation shown in Fig. 2b, the first confocal image set was acquired after 10 s of dye addition, and then acquired sequentially at 30 s intervals over 10 min. The confocal images were acquired at a position below the adhesion plane of 0–100 µm with a 10 µm interval, a size of 512 × 512 nm^2^, and a gain of 690 with no averaging.

For the short periodic observation shown in Fig. 2c, the first confocal images were acquired after 6 s of dye addition, and then acquired sequentially at 4 s interval over 50 s. Confocal images were acquired at 0, 25, 50, and 75 µm from the adhesion plane. The images were sized at 256 × 256 nm^2^, gain of 690, and pinhole of 1.0 AU (90 µm diameter for AF647) with no averaging.

### Image processing and analysis

All images were prepared using Zen software (Zeiss) by adjusting the gain and black/white balance. Structural analysis of the individual cells was performed using Neurolucida (MBF Bioscience, Williston, VT, US). Individual cell contours were picked semi-automatically from the single NCI image by Neurolucida, and the structural parameters (area, perimeter, and roundness) were calculated (Neurolucida, Figs. 1f–h, 3e–g, and Extended data Fig. 6b–d). The cell number are around 150 contour of cells per single confocal image. The histogram of the structural parameters was prepared using Excel software. The reconstructed 3D cell structures were prepared using Neurolucida from the sequential confocal tomogram from 0 to 100 µm with 2 µm interval acquisition (Fig. 1c,i insert, Fig. 3a–c insert, and Extended Data Fig. 6e). The histogram of cell volume in Figs. 1i, 3h and Extended Data Fig. 6f were prepared with the reconstructed 3D cells located in the 50 × 50 × 100 µm virtual area at the center of the scanned spheroid. The cell number were around 50 per analyzed area in single spheroid. Fluorescence intensities were analyzed using Fiji software (https://imagej.net/Fiji). The dye diffusion rate graph was prepared from the fluorescent intensity of the 50 × 50 µm virtual area at the center of the NCI image scanned at 70 µm (Fig. 2b) or 75 µm (Fig. 2c) above the adhesion plane. The drug-response change of the intercellular space shown in Fig. 3d was calculated by Fiji by subtracting the whole spheroid area of a single NCI image from the intercellular space area, and both were defined by automatic threshold setting of Fiji. The DX response fluorescence change in Fig. 4f was calculated by comparing the total fluorescence of the spheroid area in the target NCI image to the non-treated sample, set at 100%. The histogram indicating fluorescence change was obtained across the intercellular space of the spheroid and visualized with AF and the plasma membrane with AcGFP, which was temporally expressed in the cytoplasm conjugated with plasma membrane transporting signal peptide (Extended data Fig. 2); it was plotted from a single LSM image of the HEK293T spheroid and quantified using Zen software (Zeiss).

### RNA preparation

A total of 180 spheroids with or without DX treatment were collected and washed twice with PBS. The spheroids were transferred to low-adhesion microtubes and homogenized using a QIAshredder (QIAGEN, Hilden, Germany). Total RNA was harvested using an RNeasy Plus Mini Kit (Qiagen) according to the manufacturer’s protocol. The total RNA bound to the spin column was treated with recombinant DNase I (30 units, TOYOBO, Osaka, Japan) for on-column digestion for 15 min at room temperature and washed again with the accessory solution. Total RNA was eluted with 50 µL of MilliQ water, and its RNA concentration and quality were assessed by reading the absorbance at 230, 260, and 280 nm. Approximately 5 µg of total RNA was collected using this protocol.

### Transcriptomic analysis

All transcriptomic studies were performed using Novogene (Cambridge, UK). In brief, mRNA was enriched from the total RNA sample, fragmented, double-stranded cDNA synthesized, which was ligated to adaptor sets, enriched by polymerase chain reaction, and sequenced on an Illumina HiSeq (San Diego, CA, USA). Each dataset was subjected to filtering, human genome mapping, quality check, differential gene expression analysis, and gene ontology enrichment analysis (KEGG).

### Statistical analysis

The number of replicates is shown in the figure legends. Statistical significance was determined by one-way ANOVA followed by Dunnett’s test or the Tukey–Kramer test, and the P-values are shown as a respective graph. PRISM version 9.2.0 (GraphPad Software) was used for the analysis. Due to the small sample size of each experimental condition, we could not perform normality test. One-way ANOVA was performed assuming a normal distribution.

## Supplementary Information

Supplementary Information.

## Data Availability

All data obtained during study are available from the corresponding author upon request.
