# Supplementary material for: One-step visualization of natural cell activities in non-labeled living spheroids
Source: Sci Rep. 2022 Jan 27;12:1500. doi: 10.1038/s41598-022-05347-z (PMC8795241; doi:10.1038/s41598-022-05347-z)
Supplement: Supplementary file 1 — Supplementary Information. [file 41598_2022_5347_MOESM1_ESM.pdf]

# Extended Data Figure 1

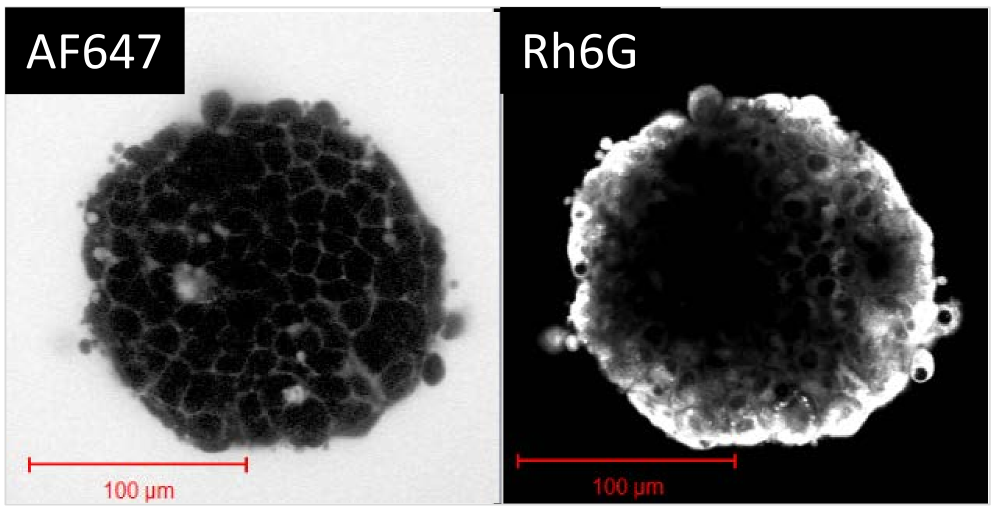


Extended Data Figure 1. The hydrophobicity of the fluorescent dye affects the inflow diffusion rate of the dye into the target spheroid. Dual-color confocal microscopy was performed for HepG2 spheroid NCI using the hydrophilic fluorescent dye AF647 (left) and the hydrophilic fluorescent dye Rhodamine 6G (Rh6G, right). One hour after the addition of the two dyes, compared to AF647, Rh6G fluorescence was observed with marked accumulation only in peripheral cells but little in the core region. Bar; 100 µm. The images were prepared by ZEN software (Zeiss, Jena, Germany).

Extended Data Figure 2

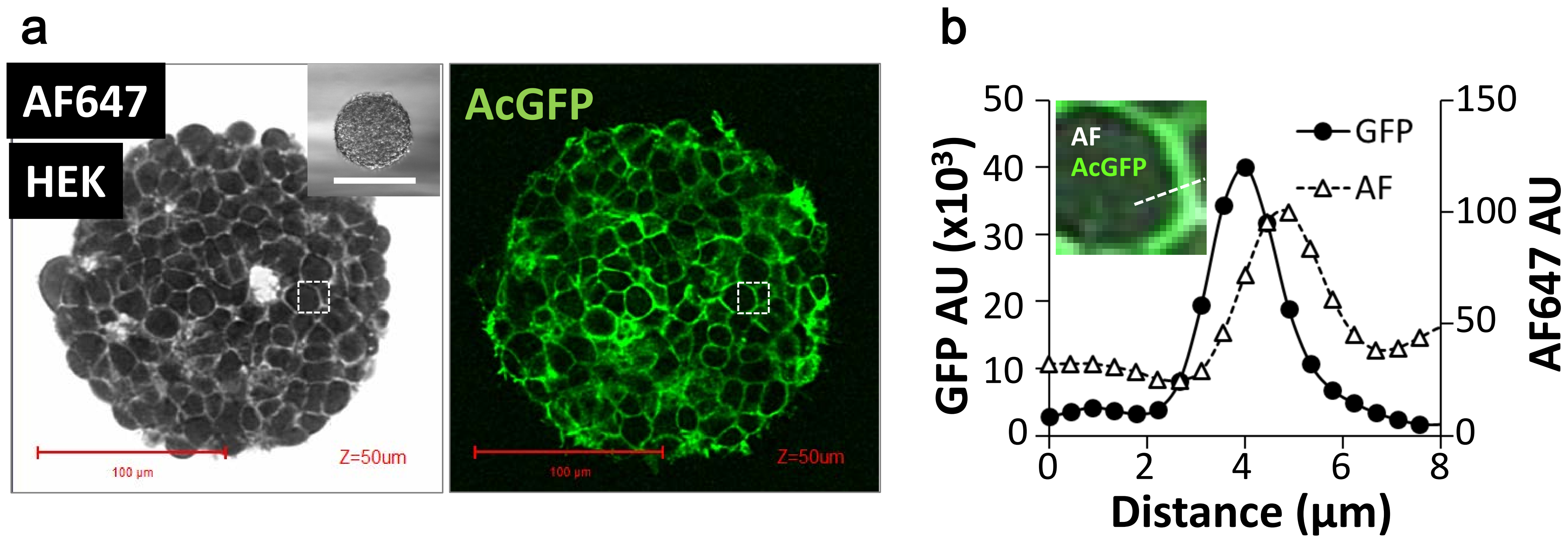


Extended Data Figure 2. NCI accurately visualizes the intercellular space of the spheroid. (a) A dual-color confocal image of a representative HEK293T (HEK) spheroid with transient expression of plasma membrane-targeting AcGFP in the cytosol. NCI with AF647 (left) and AcGFP images (right). Bar: 100 µm. Dashed rectangle: enlarged area in the inset (b). Inset: bright field. Bar: 200 µm. (b) Merged histograms of AcGFP (solid circles) and AF647 (blank triangles) fluorescence intensity along the dashed line in the inset. Inset: Merged image of the indicated dashed square in each image in (a). Square size: 16 × 16 µm. The AcGFP signal (green: intracellular side of the plasma membrane) was located beneath the AF647 signal (white: intercellular). The images were prepared by ZEN software.

Extended Data Figure 3

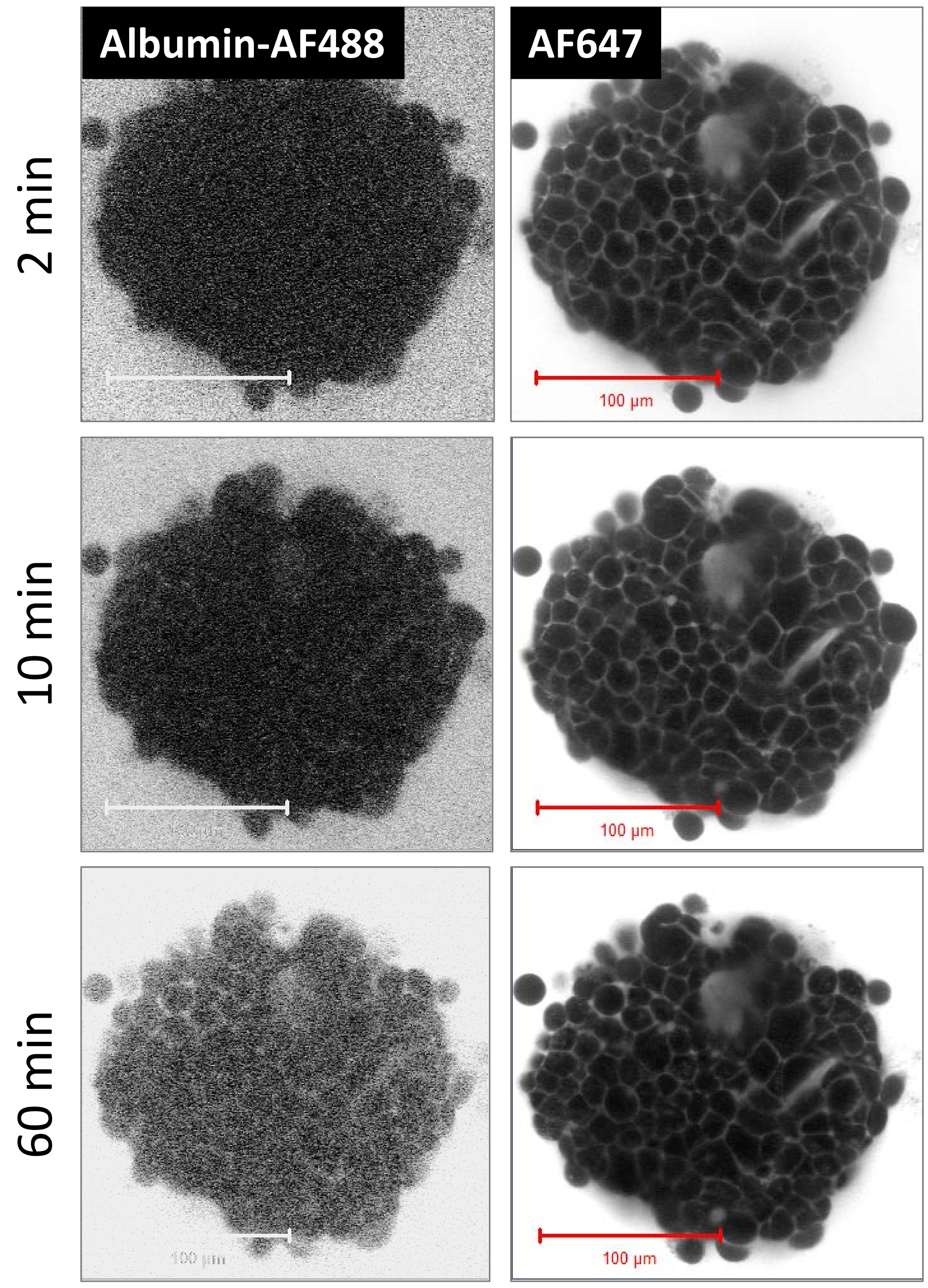


Extended Data Figure 3. The molecular weight of the dye can affect the inflow diffusion rate into the core region of the target spheroids. AF488-conjugated albumin was added to the medium of HepG2 spheroids together with AF647. Dual-color confocal imaging was performed at the same z position of the spheroid over a time course of 2, 10, and 60 min after the addition of dye. Higher molecular weight of dye yielded slower diffusion rates. Bar: 100 µm. The images were prepared by ZEN software.

Extended Data Figure 4

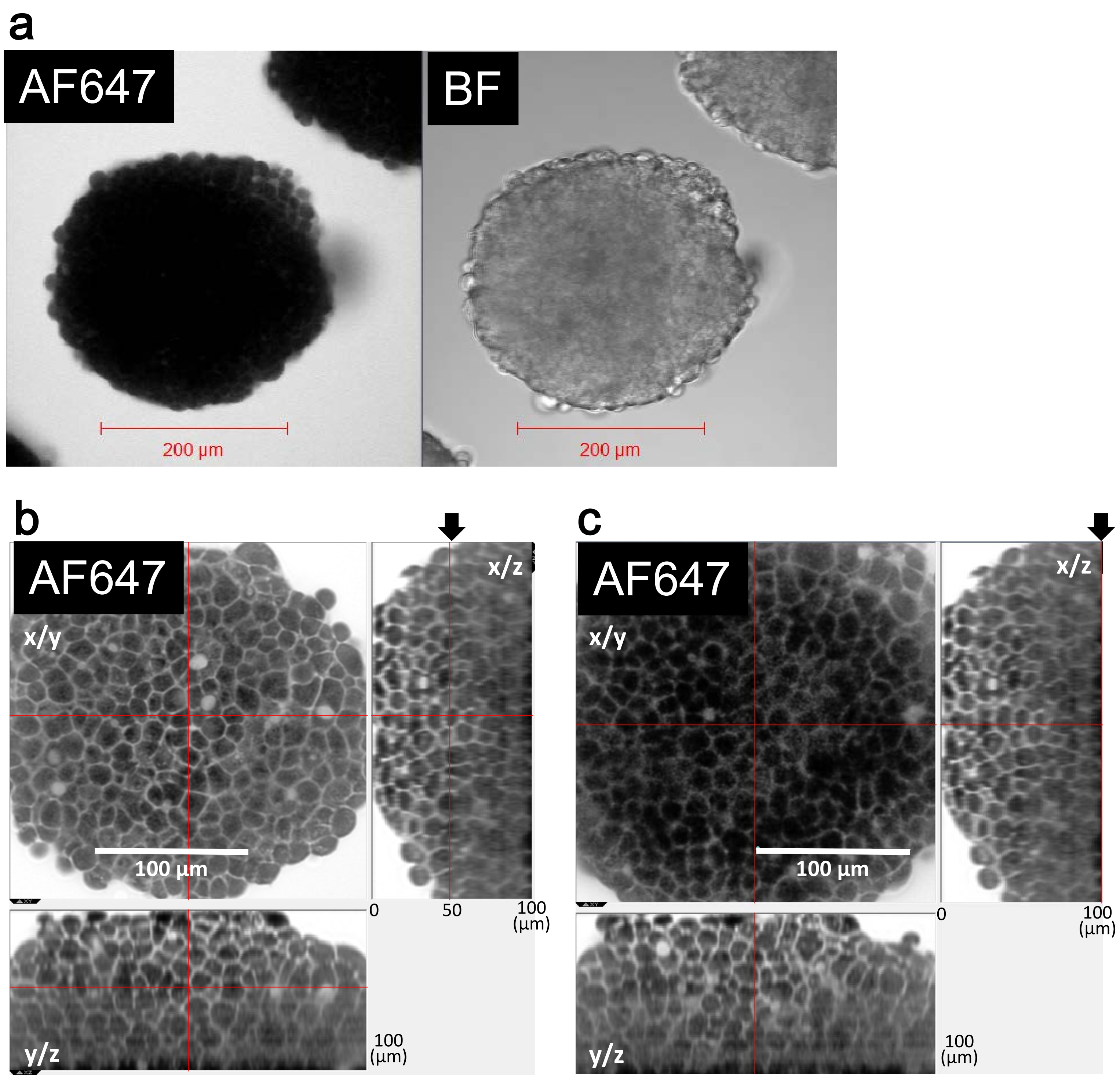


Extended Data Figure 4. NCI visualizes the cells in the deep core region of HepG2 spheroids. (a) Low-magnitude image of the HepG2 spheroid acquired by NCI (left: AF647) and bright field (right: BF). (b, c) Confocal images of the same spheroid in (a) at (b) 50 µm and (c) 100 µm distance from the adhesion plane with the orthogonal images. Bar: 100 µm. Arrow: z position of the x/y plane. Individual cells can be identified by the separation of fluorescent signals in the intercellular space. The images were prepared by ZEN software.

Extended Data Figure 5

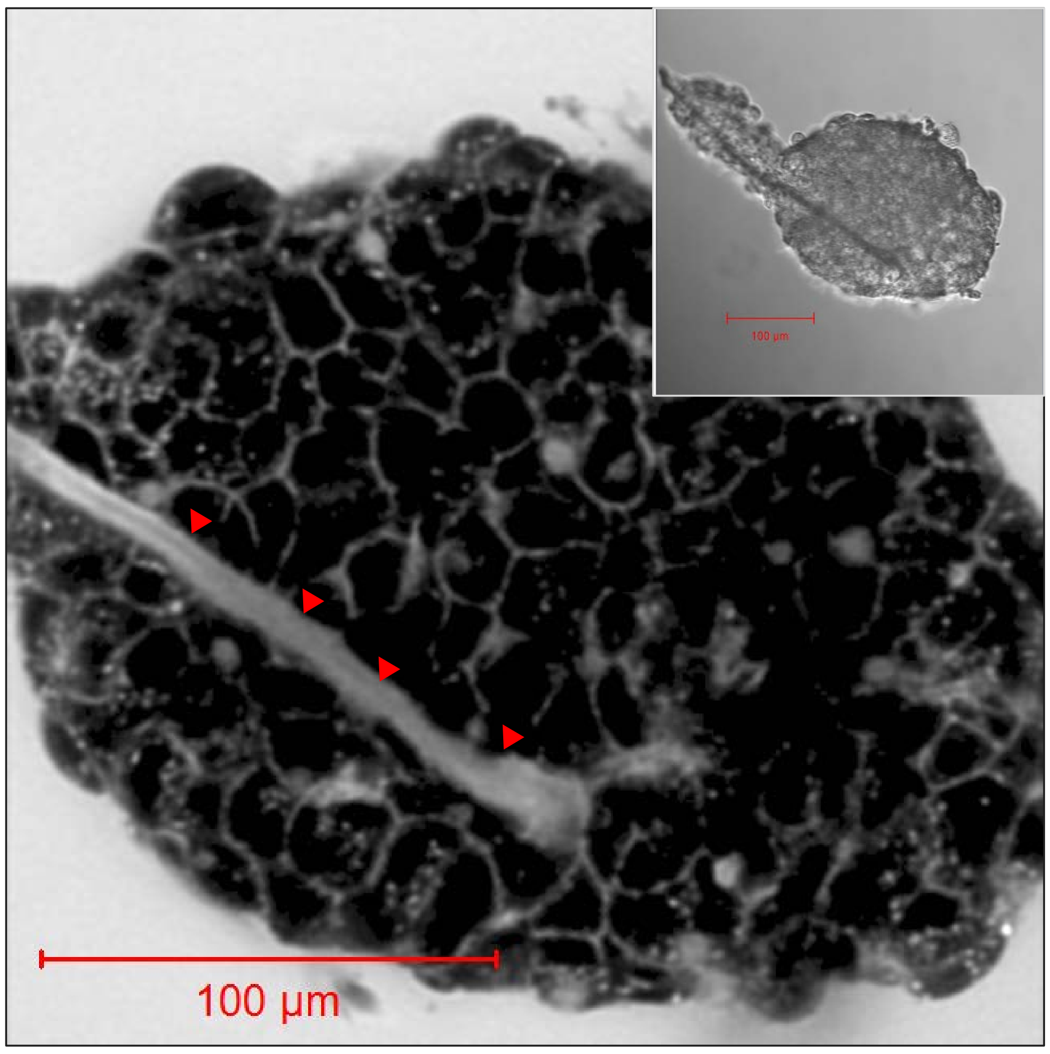


Extended Data Figure 5. NCI can visualize non-cellular insoluble components in spheroids. Representative NCI image of day 5 HepG2 spheroid that embeds insoluble filament component penetrating the dye (arrowhead), which might have originated from debris in the medium. Scale bar: 100 μm. Inset: Low-magnitude image of the spheroid in the bright field. Bar: 100 μm. The images were prepared by ZEN software.

Extended Data Figure 6

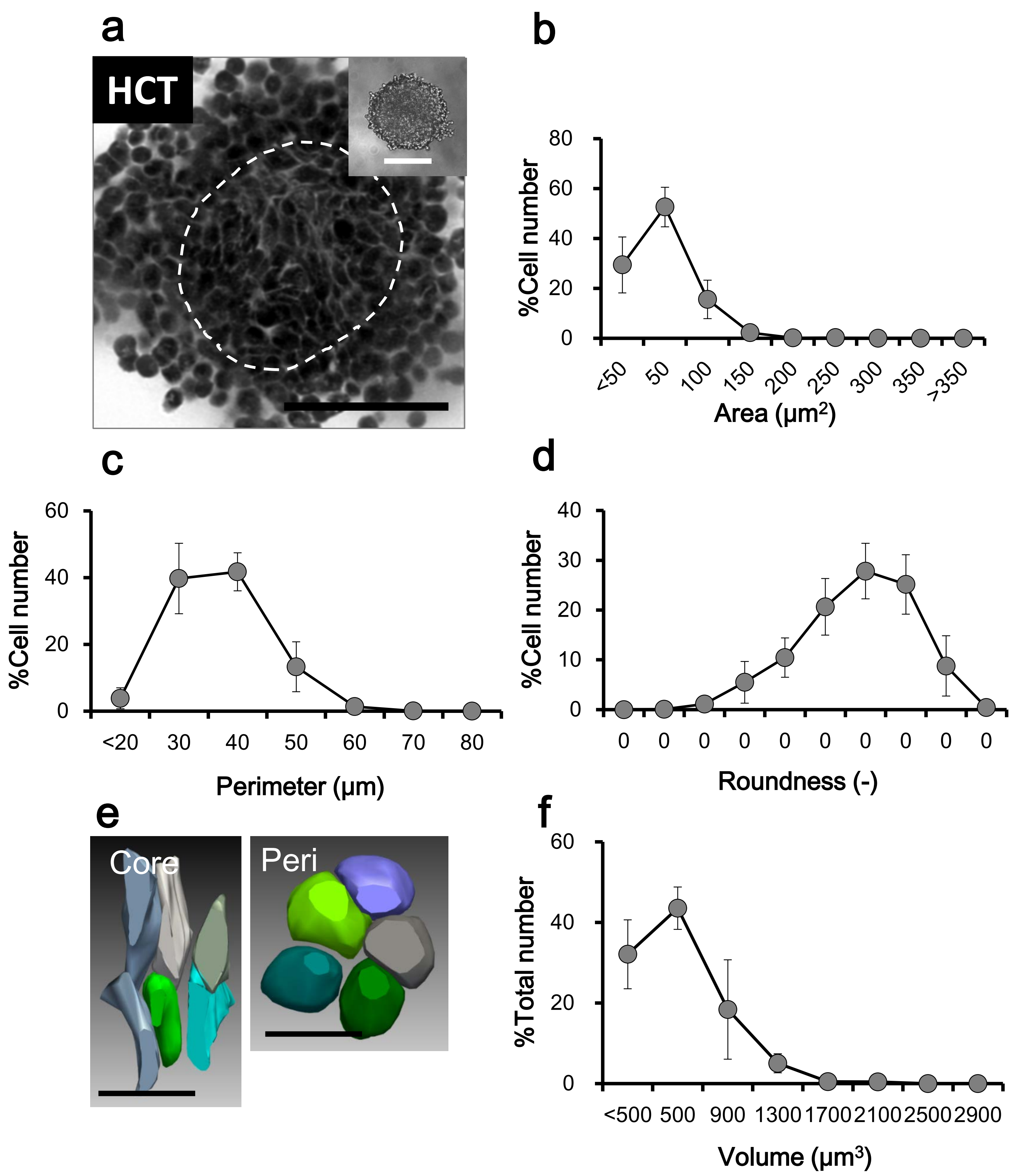


Extended Data Figure 6. NCI of a colon cancer-delivered cell line, HCT-116 (HCT). (a) Representative NCI image of HCT spheroids on day 5 of culture. The dashed line indicates the boundary between the inner tight and outer rough-cell-packaging areas. Bar: 100 µm. Inset: Bright fields. Bar: 200µm. (b-d) Histograms of the individual cellular contours in the spheroid confocal images analyzed by the (b) area, (c) perimeter, and (d) roundness. N = 4. (e) Representative images of the reconstructed 3D structures of individual core cells in HCT spheroids. Each cell is indicated by a different color. Core: core cells; Peri: peripheral cells in (a). (f) Volumetric histogram of the spheroid core cells measured in the same way as in Figure 1i. N = 3. The images of (a) were prepared by ZEN software, and the structural parameters (b-d) , 3D reconstruction of (e) and individual cell volumes of (f) were calculated by Neurolucida (MBF Bioscience, Williston, VT, US).

Extended Data Figure 7

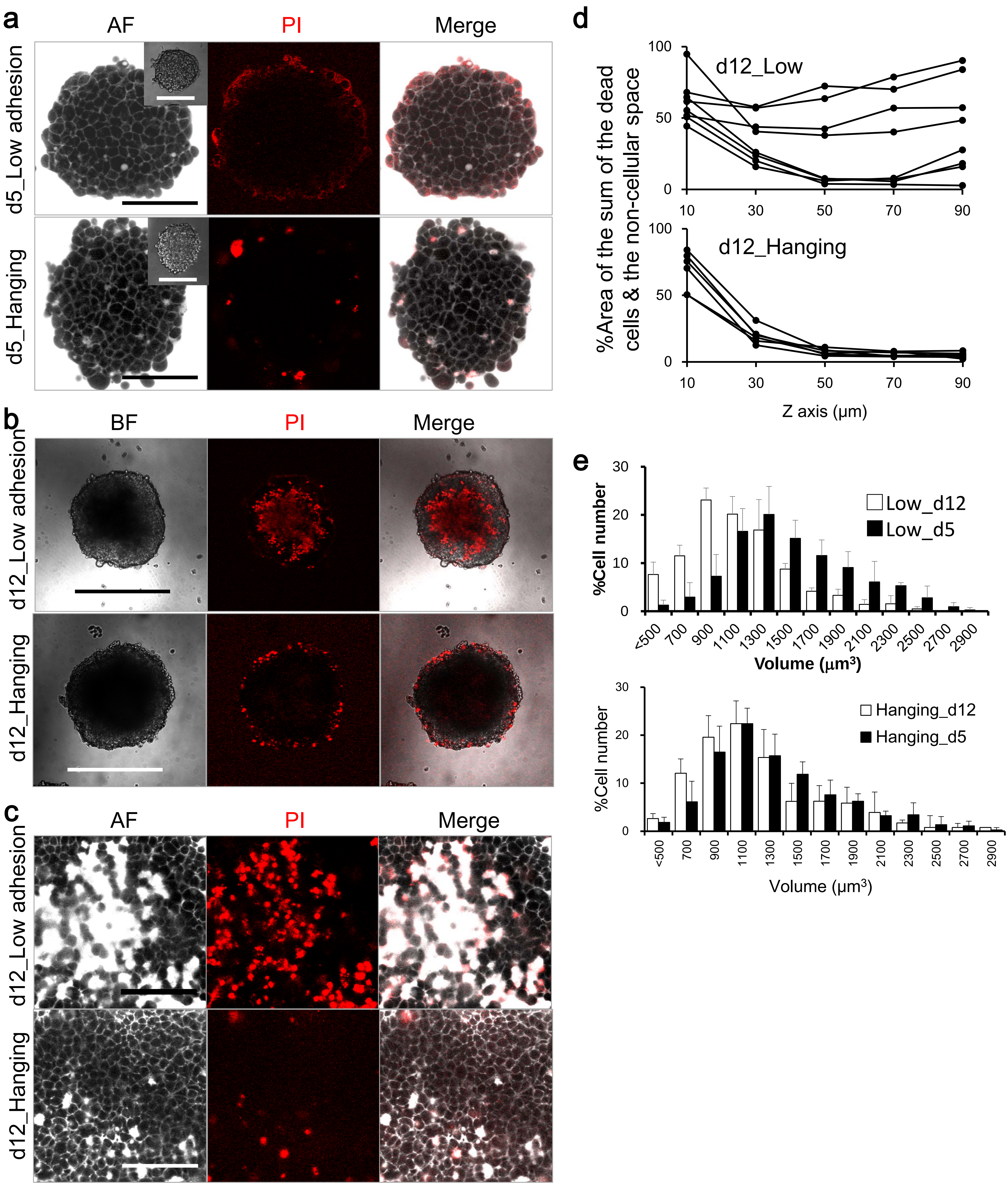

Extended Data Figure 7. NCI shows the difference in structural characteristics and viability of two spheroids cultured under different conditions. (a) HepG2 spheroids cultured for 5 days in a low-adhesion culture plate (upper) and by hanging drop method (lower) visualized by NCI (left column), propidium iodide (PI, middle), and merged (right). Bar: 100 µm. A slight difference was observed between the two spheroids. (b) HepG2 spheroids cultured under the same conditions as in (a) for 12 days. Bright field image (left), confocal image with PI (middle), and merged (right). Scale bar: 500 µm. Clear PI accumulation, which may indicate cell death, was observed in the spheroid core region, which was cultured using a low-adhesion culture plate. (c) Confocal image of the core region of spheroids (b) 100 µm above the adhesion plane. NCI (left column), PI (middle), and merged (right). Scale bar: 100 µm. (d) The content of the area of the sum of dead cells and non-cellular space against the entire scanned area aligned along the z-axis from the adhesion plane to 90 µm. Upper: low-adhesion plate. N = 8. Lower: hanging drop method. N = 6. Each line indicates individual spheroid data. (e) Volumetric histograms of individual core cells of HepG2 spheroids cultured in the low-adhesion culture plate (upper panel) and by hanging drop method (lower panel). The five-day culture data are shown in the solid column, and 12 day culture data are shown in the blank column. The low-adhesion culture plate showed a peak shift in the cell volume histogram. The images of (a-c) were prepared by ZEN software, and calculation of the fluorescence intensity in (d) were by Fiji software (https://imagej.net/Fiji). The volume of the individual cells of (e) were calculated by Neurolucida.

## Extended Data Figure 8

**a**

| Non/Mono | Non/Sphe |
|---|---|
| DX/Mono | DX/Sphe |

mRNA
-> Library (adaptor ligation)
-> PCR
-> QC
-> Seq (illumina HiSeq6000)
-> Mapping (Human genome: TopHat2)
-> Exp. Quantification (HTSeq)
-> Dif. Exp. Anal. (DESeq)
-> Norm. (DESeq)
-> FDR est. (BH)
-> GO Enrichment

**b**

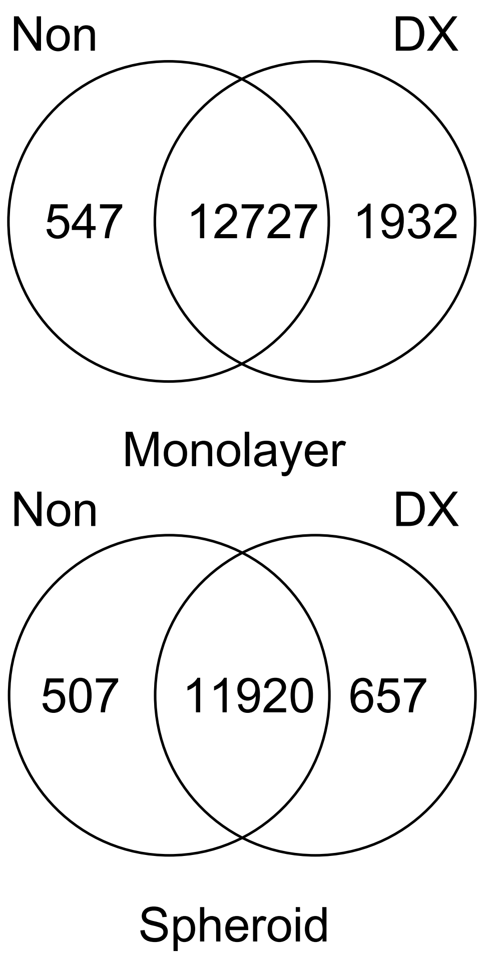


**c**

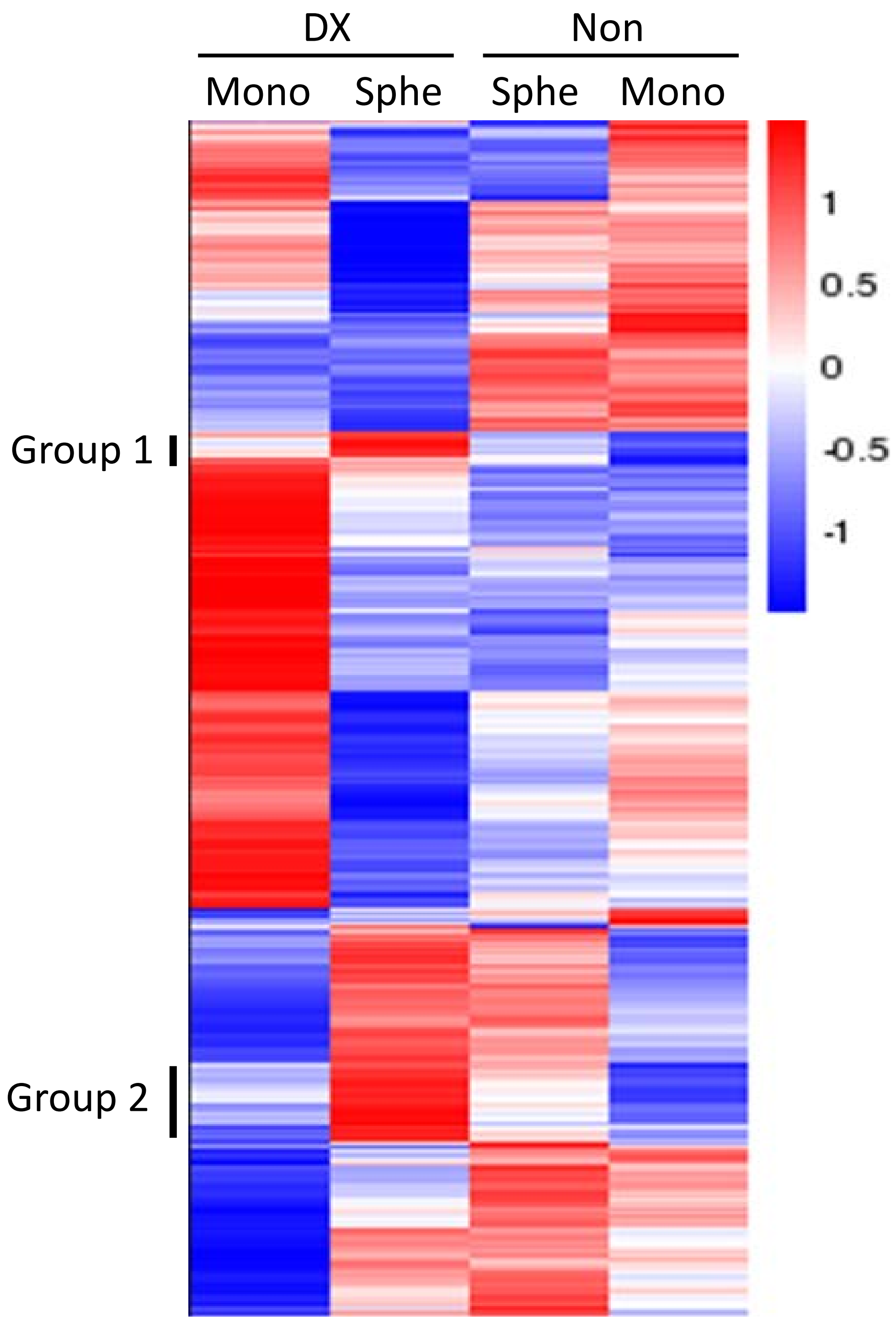


**d**

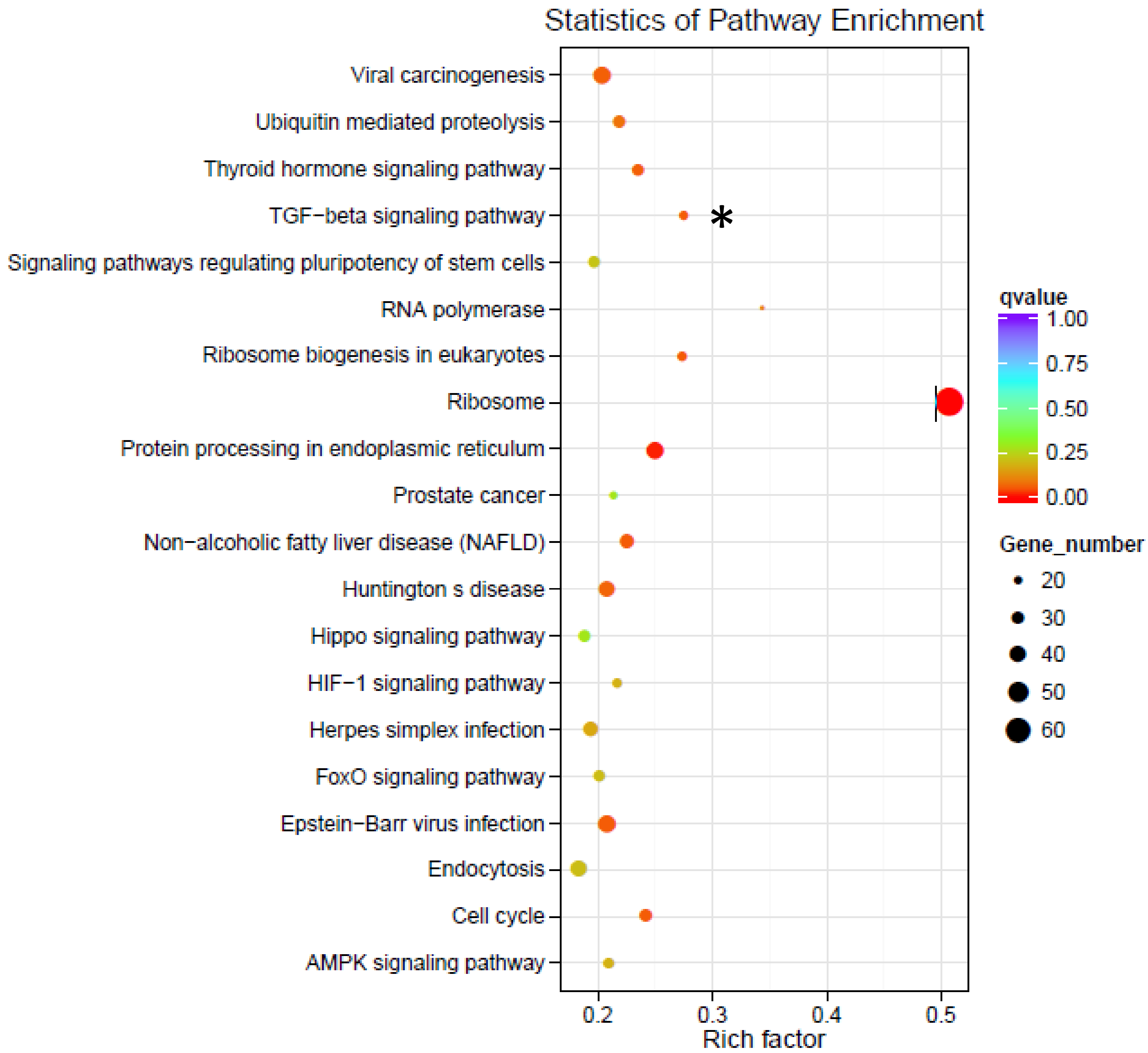

Extended Data Figure 8. Transcriptomic analysis of HepG2 cells under various culture conditions. (a) Schema of transcriptomic analysis. The four conditions in HepG2 cells cultured with monolayer (Mono) or spheroid (Sphe) with/without DX were compared. QC, quality control; FDR, false discovery rate; BH, Benjamini –Hochberg. (b) Venn diagrams representing the number of unique and common genes between the two RNA-seq samples. (c) FPKM cluster analysis (heat map) using the log10 (FPKM+1) value. The color range from red to blue represents the log10 (FPKM+1) value from large to small. Overall, 3,795 of 12,427 genes analyzed were found to be differentially expressed after multiple testing corrections (FDR estimation BH, <0.05). The upregulated gene clusters in DX-treated spheroids were named Group 1 (84 genes) and Group 2 (126 genes), and the list is shown in Extended Data Figure 9 (Group 1) and 10 (Group 2). (d) KEGG enrichment scatter plot of DX enhancement in spheroids. Dot size represents the number of different genes, and color indicates the q-value. Asterisk: TGF-β signaling pathway.

# Extended Data Figure 9

| Gene ID | Gene Name |
|---|---|
| ENSG00000137509 | prolylcarboxypeptidase(PRCP) |
| ENSG00000089063 | transmembrane protein 230(TMEM230) |
| ENSG00000156467 | ubiquinol-cytochrome c reductase binding protein(UQCRB) |
| ENSG00000105438 | KDEL endoplasmic reticulum protein retention receptor 1(KDELR1) |
| ENSG00000127184 | microRNA 3607(MIR3607) |
| ENSG00000179222 | MAGE family member D1(MAGED1) |
| ENSG00000099624 | ATP synthase, H+ transporting, mitochondrial F1 complex, delta subunit(ATP5D) |
| ENSG00000177600 | ribosomal protein lateral stalk subunit P2(RPLP2) |
| ENSG00000065978 | Y-box binding protein 1(YBX1) |
| ENSG00000139644 | transmembrane BAX inhibitor motif containing 6(TMBIM6) |
| ENSG00000111275 | aldehyde dehydrogenase 2 family (mitochondrial)(ALDH2) |
| ENSG00000166794 | peptidylprolyl isomerase B(PPIB) |
| ENSG00000142541 | ribosomal protein L13a(RPL13A) |
| ENSG00000167004 | protein disulfide isomerase family A member 3(PDIA3) |
| ENSG00000170889 | ribosomal protein S9(RPS9) |
| ENSG00000101439 | cystatin C(CST3) |
| ENSG00000163041 | H3 histone family member 3A(H3F3A) |
| ENSG00000169100 | solute carrier family 25 member 6(SLC25A6) |
| ENSG00000105193 | ribosomal protein S16(RPS16) |
| ENSG00000160014 | calmodulin 3(CALM3) |
| ENSG00000109472 | carboxypeptidase E(CPE) |
| ENSG00000130707 | argininosuccinate synthase 1(ASS1) |
| ENSG00000167526 | ribosomal protein L13(RPL13) |
| ENSG00000143870 | protein disulfide isomerase family A member 6(PDIA6) |
| ENSG00000166710 | beta-2-microglobulin(B2M) |
| ENSG00000160213 | cystatin B(CSTB) |
| ENSG00000155368 | diazepam binding inhibitor, acyl-CoA binding protein(DBI) |
| ENSG00000161944 | asialoglycoprotein receptor 2(ASGR2) |
| ENSG00000138207 | retinol binding protein 4(RBP4) |
| ENSG00000197249 | serpin family A member 1(SERPINA1) |
| ENSG00000198931 | adenine phosphoribosyltransferase(APRT) |
| ENSG00000170540 | ADP ribosylation factor like GTPase 6 interacting protein 1(ARL6IP1) |
| ENSG00000115268 | ribosomal protein S15(RPS15) |
| ENSG00000110492 | midkine (neurite growth-promoting factor 2)(MDK) |
| ENSG00000136942 | ribosomal protein L35(RPL35) |
| ENSG00000132386 | serpin family F member 1(SERPINF1) |
| ENSG00000173457 | protein phosphatase 1 regulatory inhibitor subunit 14B(PPP1R14B) |
| ENSG00000233927 | ribosomal protein S28(RPS28) |
| ENSG00000120885 | microRNA 6843(MIR6843) |
| ENSG00000221983 | ubiquitin A-52 residue ribosomal protein fusion product 1(UBA52) |
| ENSG00000162244 | ribosomal protein L29(RPL29) |
| ENSG00000142534 | ribosomal protein S11(RPS11) |
| ENSG00000109861 | cathepsin C(CTSC) |
| ENSG00000149273 | ribosomal protein S3(RPS3) |
| ENSG00000153574 | ribose 5-phosphate isomerase A(RPIA) |
| ENSG00000065154 | ornithine aminotransferase(OAT) |
| ENSG00000112306 | ribosomal protein S12(RPS12) |
| ENSG00000136156 | integral membrane protein 2B(ITM2B) |
| ENSG00000135744 | angiotensinogen(AGT) |
| ENSG00000158104 | 4-hydroxyphenylpyruvate dioxygenase(HPD) |
| ENSG00000131143 | cytochrome c oxidase subunit 4I1(COX4I1) |
| ENSG00000117632 | microRNA 3917(MIR3917) |
| ENSG00000135940 | cytochrome c oxidase subunit 5B(COX5B) |
| ENSG00000118137 | apolipoprotein A1(APOA1) |
| ENSG00000116221 | mitochondrial ribosomal protein L37(MRPL37) |
| ENSG00000125827 | thioredoxin related transmembrane protein 4(TMX4) |
| ENSG00000159199 | ATP synthase, H+ transporting, mitochondrial Fo complex subunit C1 (subunit 9)(ATP5G1) |
| ENSG00000186468 | ribosomal protein S23(RPS23) |
| ENSG00000063177 | ribosomal protein L18(RPL18) |
| ENSG00000140988 | ribosomal protein S2(RPS2) |
| ENSG00000147257 | glypican 3(GPC3) |
| ENSG00000129824 | ribosomal protein S4, Y-linked 1(RPS4Y1) |
| ENSG00000147255 | immunoglobulin superfamily member 1(IGSF1) |
| ENSG00000105372 | ribosomal protein S19(RPS19) |
| ENSG00000124299 | peptidase D(PEPD) |
| ENSG00000161203 | adaptor related protein complex 2 mu 1 subunit(AP2M1) |
| ENSG00000093010 | microRNA 4761(MIR4761) |
| ENSG00000137818 | ribosomal protein lateral stalk subunit P1(RPLP1) |
| ENSG00000178445 | glycine decarboxylase(GLDC) |
| ENSG00000163902 | ribophorin I(RPN1) |
| ENSG00000155660 | protein disulfide isomerase family A member 4(PDIA4) |
| ENSG00000083845 | ribosomal protein S5(RPS5) |
| ENSG00000169174 | proprotein convertase subtilisin/kexin type 9(PCSK9) |
| ENSG00000243147 | mitochondrial ribosomal protein L33(MRPL33) |
| ENSG00000244038 | dolichyl-diphosphooligosaccharide--protein glycosyltransferase non-catalytic subunit(DDOST) |
| ENSG00000177556 | antioxidant 1 copper chaperone(ATOX1) |
| ENSG00000175899 | alpha-2-macroglobulin(A2M) |
| ENSG00000167996 | ferritin heavy chain 1(FTH1) |
| ENSG00000206503 | major histocompatibility complex, class I, A(HLA-A) |
| ENSG00000125995 | reactive oxygen species modulator 1(ROMO1) |
| ENSG00000101444 | adenosylhomocysteinase(AHCY) |
| ENSG00000123131 | peroxiredoxin 4(PRDX4) |
| ENSG00000149547 | EI24, autophagy associated transmembrane protein(EI24) |
| ENSG00000105640 | ribosomal protein L18a(RPL18A) |

Extended Data Figure 9. The contents of Group 1 of the heat map shown in Extended Data Fig. 8b.

# Extended Data Figure 10

| Gene ID | Gene Name | Gene ID | Gene Name |
|---|---|---|---|
| ENSG00000129562 | defender against cell death 1(DAD1) | ENSG00000198034 | ribosomal protein S4, X-linked(RPS4X) |
| ENSG00000134910 | STT3A, catalytic subunit of the oligosaccharyltransferase complex(STT3A) | ENSG00000118181 | ribosomal protein S25(RPS25) |
| ENSG00000107537 | phytanoyl-CoA 2-hydroxylase(PHYH) | ENSG00000106538 | retinoic acid receptor responder 2(RARRES2) |
| ENSG00000110700 | ribosomal protein S13(RPS13) | ENSG00000008988 | ribosomal protein S20(RPS20) |
| ENSG00000116005 | prenylcysteine oxidase 1(PCYOX1) | ENSG00000115128 | splicing factor 3b subunit 6(SF3B6) |
| ENSG00000117450 | peroxiredoxin 1(PRDX1) | ENSG00000197747 | S100 calcium binding protein A10(S100A10) |
| ENSG00000123349 | prefoldin subunit 5(PFDN5) | ENSG00000145425 | ribosomal protein S3A(RPS3A) |
| ENSG00000184500 | protein S (alpha)(PROS1) | ENSG00000147604 | ribosomal protein L7(RPL7) |
| ENSG00000143198 | microsomal glutathione S-transferase 3(MGST3) | ENSG00000114391 | ribosomal protein L24(RPL24) |
| ENSG00000099937 | serpin family D member 1(SERPIND1) | ENSG00000100316 | ribosomal protein L3(RPL3) |
| ENSG00000089220 | phosphatidylethanolamine binding protein 1(PEBP1) | ENSG00000185088 | ribosomal protein S27 like(RPS27L) |
| ENSG00000147677 | eukaryotic translation initiation factor 3 subunit H(EIF3H) | ENSG00000131828 | pyruvate dehydrogenase (lipoamide) alpha 1(PDHA1) |
| ENSG00000178741 | cytochrome c oxidase subunit 5A(COX5A) | ENSG00000143486 | eukaryotic translation initiation factor 2D(EIF2D) |
| ENSG00000126067 | proteasome subunit beta 2(PSMB2) | ENSG00000166228 | pterin-4 alpha-carbinolamine dehydratase 1(PCBD1) |
| ENSG00000113648 | H2A histone family member Y(H2AFY) | ENSG00000074800 | enolase 1(ENO1) |
| ENSG00000111229 | actin related protein 2/3 complex subunit 3(ARPC3) | ENSG00000106049 | 3-hydroxyisobutyrate dehydrogenase(HIBADH) |
| ENSG00000132507 | eukaryotic translation initiation factor 5A(EIF5A) | ENSG00000172590 | mitochondrial ribosomal protein L52(MRPL52) |
| ENSG00000143183 | transmembrane and coiled-coil domains 1(TMCO1) | ENSG00000114902 | signal peptidase complex subunit 1(SPCS1) |
| ENSG00000130208 | apolipoprotein C1(APOC1) | ENSG00000142937 | ribosomal protein S8(RPS8) |
| ENSG00000118680 | myosin light chain 12B(MYL12B) | ENSG00000196683 | translocase of outer mitochondrial membrane 7(TOMM7) |
| ENSG00000173852 | dpy-19 like 1(DPY19L1) | ENSG00000118271 | transthyretin(TTR) |
| ENSG00000116478 | histone deacetylase 1(HDAC1) | ENSG00000099783 | heterogeneous nuclear ribonucleoprotein M(HNRNPM) |
| ENSG00000171314 | phosphoglycerate mutase 1(PGAM1) | ENSG00000173207 | CDC28 protein kinase regulatory subunit 1B(CKS1B) |
| ENSG00000143621 | interleukin enhancer binding factor 2(ILF2) | ENSG00000136930 | proteasome subunit beta 7(PSMB7) |
| ENSG00000168028 | ribosomal protein SA(RPSA) | ENSG00000172115 | cytochrome c, somatic(CYCS) |
| ENSG00000114054 | propionyl-CoA carboxylase beta subunit(PCCB) | ENSG00000168653 | NADH:ubiquinone oxidoreductase subunit S5(NDUFS5) |
| ENSG00000144713 | ribosomal protein L32(RPL32) | ENSG00000117054 | acyl-CoA dehydrogenase, C-4 to C-12 straight chain(ACADM) |
| ENSG00000128928 | isovaleryl-CoA dehydrogenase(IVD) | ENSG00000165264 | NADH:ubiquinone oxidoreductase subunit B6(NDUFB6) |
| ENSG00000108298 | ribosomal protein L19(RPL19) | ENSG00000100983 | glutathione synthetase(GSS) |
| ENSG00000182551 | acireductone dioxygenase 1(ADI1) | ENSG00000126457 | protein arginine methyltransferase 1(PRMT1) |
| ENSG00000142657 | phosphogluconate dehydrogenase(PGD) | ENSG00000086232 | eukaryotic translation initiation factor 2 alpha kinase 1(EIF2AK1) |
| ENSG00000081051 | alpha fetoprotein(AFP) | ENSG00000175061 | LRRC75A antisense RNA 1(LRRC75A-AS1) |
| ENSG00000164587 | ribosomal protein S14(RPS14) | ENSG00000214078 | copine 1(CPNE1) |
| ENSG00000124562 | small nuclear ribonucleoprotein polypeptide C(SNRPC) | ENSG00000138326 | ribosomal protein S24(RPS24) |
| ENSG00000204308 | ring finger protein 5(RNF5) | ENSG00000119013 | NADH:ubiquinone oxidoreductase subunit B3(NDUFB3) |
| ENSG00000122026 | ribosomal protein L21(RPL21) | ENSG00000117601 | serpin family C member 1(SERPINC1) |
| ENSG00000165678 | growth hormone inducible transmembrane protein(GHITM) | ENSG00000174547 | mitochondrial ribosomal protein L11(MRPL11) |
| ENSG00000107798 | lipase A, lysosomal acid type(LIPA) | ENSG00000106153 | coiled-coil-helix-coiled-coil-helix domain containing 2(CHCHD2) |
| ENSG00000164904 | aldehyde dehydrogenase 7 family member A1(ALDH7A1) | ENSG00000131469 | ribosomal protein L27(RPL27) |
| ENSG00000130414 | NADH:ubiquinone oxidoreductase subunit A10(NDUFA10) | ENSG00000104325 | 2,4-dienoyl-CoA reductase 1, mitochondrial(DECR1) |
| ENSG00000113558 | S-phase kinase associated protein 1(SKP1) | ENSG00000110321 | eukaryotic translation initiation factor 4 gamma 2(EIF4G2) |
| ENSG00000145592 | ribosomal protein L37(RPL37) | ENSG00000025772 | translocase of outer mitochondrial membrane 34(TOMM34) |
| ENSG00000171560 | fibrinogen alpha chain(FGA) | ENSG00000137563 | gamma-glutamyl hydrolase(GGH) |
| ENSG00000196502 | sulfotransferase family 1A member 1(SULT1A1) | ENSG00000132432 | Sec61 translocon gamma subunit(SEC61G) |
| ENSG00000058262 | Sec61 translocon alpha 1 subunit(SEC61A1) | ENSG00000130255 | ribosomal protein L36(RPL36) |
| ENSG00000164111 | annexin A5(ANXA5) | ENSG00000134291 | transmembrane protein 106C(TMEM106C) |
| ENSG00000230989 | heat shock factor binding protein 1(HSBP1) | ENSG00000229117 | ribosomal protein L41(RPL41) |
| ENSG00000125868 | destrin, actin depolymerizing factor(DSTN) | ENSG00000150779 | translocase of inner mitochondrial membrane 8 homolog B(TIMM8B) |
| ENSG00000117592 | peroxiredoxin 6(PRDX6) | ENSG00000051596 | THO complex 3(THOC3) |
| ENSG00000125743 | small nuclear ribonucleoprotein D2 polypeptide(SNRPD2) | ENSG00000172809 | ribosomal protein L38(RPL38) |
| ENSG00000182220 | ATPase H+ transporting accessory protein 2(ATP6AP2) | ENSG00000102144 | phosphoglycerate kinase 1(PGK1) |
| ENSG00000127922 | uncharacterized LOC100506136(LOC100506136) | ENSG00000215021 | prohibitin 2(PHB2) |
| ENSG00000164919 | cytochrome c oxidase subunit 6C(COX6C) | ENSG00000140740 | ubiquinol-cytochrome c reductase core protein II(UQCRC2) |
| ENSG00000110955 | ATP synthase, H+ transporting, mitochondrial F1 complex, beta polypeptide(ATP5B) | ENSG00000188846 | ribosomal protein L14(RPL14) |
| ENSG00000117906 | reticulocalbin 2(RCN2) | ENSG00000127884 | enoyl-CoA hydratase, short chain 1(ECHS1) |
| ENSG00000133935 | chromosome 14 open reading frame 1(C14orf1) | ENSG00000177156 | transaldolase 1(TALDO1) |
| ENSG00000138029 | hydroxyacyl-CoA dehydrogenase/3-ketoacyl-CoA thiolase/enoyl-CoA hydratase (trifunctional protein), beta subunit(HADHB) | ENSG00000131238 | palmitoyl-protein thioesterase 1(PPT1) |
| ENSG00000136521 | NADH:ubiquinone oxidoreductase subunit B5(NDUFB5) | ENSG00000112977 | death associated protein(DAP) |
| ENSG00000196975 | annexin A4(ANXA4) | ENSG00000111640 | glyceraldehyde-3-phosphate dehydrogenase(GAPDH) |
| ENSG00000136888 | ATPase H+ transporting V1 subunit G1(ATP6V1G1) | ENSG00000174444 | ribosomal protein L4(RPL4) |
| ENSG00000112695 | cytochrome c oxidase subunit 7A2(COX7A2) | ENSG00000173113 | tRNA methyltransferase 11-2 homolog (S. cerevisiae)(TRMT112) |
| ENSG00000116251 | ribosomal protein L22(RPL22) | ENSG00000198755 | ribosomal protein L10a(RPL10A) |
| ENSG00000184076 | ubiquinol-cytochrome c reductase, complex III subunit X(UQCR10) | ENSG00000228278 | orosomucoid 2(ORM2) |
| ENSG00000143162 | cellular repressor of E1A stimulated genes 1(CREG1) | ENSG00000177954 | ribosomal protein S27(RPS27) |
| ENSG00000147400 | centrin 2(CETN2) | ENSG00000071082 | ribosomal protein L31(RPL31) |
| ENSG00000147403 | ribosomal protein L10(RPL10) | ENSG00000117862 | thioredoxin domain containing 12(TXNDC12) |
| ENSG00000187109 | nucleosome assembly protein 1 like 1(NAP1L1) | ENSG00000115561 | charged multivesicular body protein 3(CHMP3) |
| ENSG00000213741 | uncharacterized LOC100288910(LOC100288910) | ENSG00000135390 | ATP synthase, H+ transporting, mitochondrial Fo complex subunit C2 (subunit 9)(ATP5G2) |
| ENSG00000075415 | solute carrier family 25 member 3(SLC25A3) | ENSG00000099194 | stearoyl-CoA desaturase(SCD) |
| ENSG00000131844 | methylcrotonoyl-CoA carboxylase 2(MCCC2) | ENSG00000122406 | ribosomal protein L5(RPL5) |
| ENSG00000069535 | monoamine oxidase B(MAOB) | ENSG00000257017 | haptoglobin(HP) |
| ENSG00000103811 | cathepsin H(CTSH) | ENSG00000089157 | ribosomal protein lateral stalk subunit P0(RPLP0) |
| ENSG00000211445 | glutathione peroxidase 3(GPX3) | ENSG00000184117 | nipsnap homolog 1 (C. elegans)(NIPSNAP1) |
| ENSG00000140319 | signal recognition particle 14(SRP14) | ENSG00000125356 | NADH:ubiquinone oxidoreductase subunit A1(NDUFA1) |
| ENSG00000114942 | eukaryotic translation elongation factor 1 beta 2(EEF1B2) | ENSG00000115758 | ornithine decarboxylase 1(ODC1) |
| ENSG00000167085 | prohibitin(PHB) | ENSG00000106803 | Sec61 translocon beta subunit(SEC61B) |
| ENSG00000104904 | ornithine decarboxylase antizyme 1(OAZ1) | ENSG00000156508 | eukaryotic translation elongation factor 1 alpha 1(EEF1A1) |
| ENSG00000044115 | catenin alpha 1(CTNNA1) | ENSG00000116288 | Parkinsonism associated deglycase(PARK7) |
| ENSG00000005022 | solute carrier family 25 member 5(SLC25A5) | ENSG00000183291 | selenoprotein F(SELENOF) |
| ENSG00000109475 | ribosomal protein L34(RPL34) | ENSG00000119326 | catenin alpha like 1(CTNNAL1) |
| ENSG00000070756 | microRNA 7705(MIR7705) | ENSG00000173915 | microRNA 1307(MIR1307) |
| ENSG00000181019 | NAD(P)H quinone dehydrogenase 1(NQO1) | ENSG00000171858 | ribosomal protein S21(RPS21) |
| ENSG00000087086 | ferritin light chain(FTL) | ENSG00000164096 | chromosome 4 open reading frame 3(C4orf3) |
| ENSG00000103363 | transcription elongation factor B subunit 2(TCEB2) | ENSG00000125691 | ribosomal protein L23(RPL23) |
| ENSG00000002549 | leucine aminopeptidase 3(LAP3) | ENSG00000130005 | guanidinoacetate N-methyltransferase(GAMT) |
| ENSG00000214026 | mitochondrial ribosomal protein L23(MRPL23) | ENSG00000132541 | reactive intermediate imine deaminase A homolog(RIDA) |
| ENSG00000197043 | annexin A6(ANXA6) | ENSG00000005893 | lysosomal associated membrane protein 2(LAMP2) |
| ENSG00000068697 | lysosomal protein transmembrane 4 alpha(LAPTM4A) | ENSG00000133872 | store-operated calcium entry associated regulatory factor(SARAF) |
| ENSG00000111666 | choline phosphotransferase 1(CHPT1) | ENSG00000138760 | scavenger receptor class B member 2(SCARB2) |
| ENSG00000111669 | triosephosphate isomerase 1(TPI1) | ENSG00000143549 | tropomyosin 3(TPM3) |
| ENSG00000072274 | transferrin receptor(TFRC) | ENSG00000231500 | ribosomal protein S18(RPS18) |
| ENSG00000111786 | serine and arginine rich splicing factor 9(SRSF9) | ENSG00000182199 | serine hydroxymethyltransferase 2(SHMT2) |
| ENSG00000135776 | ATP binding cassette subfamily B member 10(ABCB10) | ENSG00000051620 | heme binding protein 2(HEBP2) |
| ENSG00000133112 | tumor protein, translationally-controlled 1(TPT1) | ENSG00000135929 | cytochrome P450 family 27 subfamily A member 1(CYP27A1) |
| ENSG00000165475 | crystallin lambda 1(CRYL1) | ENSG00000204628 | receptor for activated C kinase 1(RACK1) |
| ENSG00000197958 | ribosomal protein L12(RPL12) | ENSG00000182899 | ribosomal protein L35a(RPL35A) |
| ENSG00000060971 | acetyl-CoA acyltransferase 1(ACAA1) | ENSG00000092010 | proteasome activator subunit 1(PSME1) |
| ENSG00000137154 | ribosomal protein S6(RPS6) | ENSG00000172172 | mitochondrial ribosomal protein L13(MRPL13) |
| ENSG00000100897 | DDB1 and CUL4 associated factor 11(DCAF11) | ENSG00000104763 | N-acylsphingosine amidohydrolase 1(ASAH1) |
| ENSG00000166441 | ribosomal protein L27a(RPL27A) | ENSG00000075239 | acetyl-CoA acetyltransferase 1(ACAT1) |
| ENSG00000143947 | ribosomal protein S27a(RPS27A) | ENSG00000196262 | peptidylprolyl isomerase A(PPIA) |
| ENSG00000125970 | RALY heterogeneous nuclear ribonucleoprotein(RALY) | ENSG00000176890 | thymidylate synthetase(TYMS) |
| ENSG00000175390 | eukaryotic translation initiation factor 3 subunit F(EIF3F) | ENSG00000111058 | acyl-CoA synthetase short-chain family member 3(ACSS3) |
| ENSG00000125971 | dynein light chain roadblock-type 1(DYNLRB1) | ENSG00000130332 | LSM7 homolog, U6 small nuclear RNA and mRNA degradation associated(LSM7) |
| ENSG00000134779 | tubulin polyglutamylase complex subunit 2(TPGS2) | ENSG00000254999 | BRICK1, SCAR/WAVE actin nucleating complex subunit(BRK1) |
| ENSG00000124172 | ATP synthase, H+ transporting, mitochondrial F1 complex, epsilon subunit(ATP5E) | ENSG00000189056 | reelin(RELN) |
| ENSG00000135624 | chaperonin containing TCP1 subunit 7(CCT7) | ENSG00000084754 | hydroxyacyl-CoA dehydrogenase/3-ketoacyl-CoA thiolase/enoyl-CoA hydratase (trifunctional protein), alpha subunit(HADHA) |
| ENSG00000173660 | ubiquinol-cytochrome c reductase hinge protein(UQCRH) | ENSG00000137880 | GTP cyclohydrolase I feedback regulator(GCHFR) |
| ENSG00000112667 | 2'-deoxynucleoside 5'-phosphate N-hydrolase 1(DNPH1) | ENSG00000169567 | histidine triad nucleotide binding protein 1(HINT1) |
| ENSG00000175166 | proteasome 26S subunit, non-ATPase 2(PSMD2) | ENSG00000229314 | orosomucoid 1(ORM1) |
| ENSG00000110245 | apolipoprotein C3(APOC3) | ENSG00000090621 | poly(A) binding protein cytoplasmic 4(PABPC4) |
| ENSG00000197635 | dipeptidyl peptidase 4(DPP4) | ENSG00000163586 | fatty acid binding protein 1(FABP1) |
| ENSG00000197756 | ribosomal protein L37a(RPL37A) | ENSG00000163466 | actin related protein 2/3 complex subunit 2(ARPC2) |
| ENSG00000151552 | quinoid dihydropteridine reductase(QDPR) | ENSG00000148303 | ribosomal protein L7a(RPL7A) |
| ENSG00000198848 | carboxylesterase 1(CES1) | ENSG00000077549 | capping actin protein of muscle Z-line beta subunit(CAPZB) |
| ENSG00000142168 | superoxide dismutase 1, soluble(SOD1) | ENSG00000064115 | transmembrane 7 superfamily member 3(TM7SF3) |
| ENSG00000196419 | X-ray repair cross complementing 6(XRCC6) | ENSG00000175334 | barrier to autointegration factor 1(BANF1) |
| ENSG00000165487 | mitochondrial calcium uptake 2(MICU2) | ENSG00000171530 | tubulin folding cofactor A(TBCA) |
| ENSG00000088832 | microRNA 6869(MIR6869) | ENSG00000140374 | electron transfer flavoprotein alpha subunit(ETFA) |
| ENSG00000089009 | ribosomal protein L6(RPL6) | ENSG00000203875 | small nucleolar RNA host gene 5(SNHG5) |
| ENSG00000143252 | succinate dehydrogenase complex subunit C(SDHC) | ENSG00000084623 | eukaryotic translation initiation factor 3 subunit I(EIF3I) |
| ENSG00000149476 | triokinase and FMN cyclase(TKFC) | ENSG00000189043 | NDUFA4, mitochondrial complex associated(NDUFA4) |
| ENSG00000105202 | fibrillarin(FBL) | ENSG00000168243 | G protein subunit gamma 4(GNG4) |
| ENSG00000047634 | sex comb on midleg-like 1 (Drosophila)(SCML1) | ENSG00000006042 | transmembrane protein 98(TMEM98) |
| ENSG00000102172 | spermine synthase(SMS) | ENSG00000142676 | ribosomal protein L11(RPL11) |
| ENSG00000172340 | succinate-CoA ligase GDP-forming beta subunit(SUCLG2) | ENSG00000107223 | endothelial differentiation related factor 1(EDF1) |



Extended Data Figure 10. The contents of Group 2 of the heat map shown in Extended Data Fig. 8b.

# Extended Data Figure 11

| | Gene | FPKM Non_Sphe | Dx_Sphe | Non_Mono | Dx_Mono |
|---|---|---|---|---|---|
| Integrin | ITGA1 | 13 | 16 | 19 | 19 |
| | ITGA2 | 10 | 4 | 22 | 10 |
| | ITGA2B | 0 | 1 | 0 | 13 |
| | ITGA3 | 1 | 1 | 3 | 6 |
| | ITGA4 | 0 | 0 | 0 | 0 |
| | ITGA5 | 0 | 0 | 0 | 1 |
| | ITGA6 | 33 | 27 | 26 | 24 |
| | ITGA7 | 2 | 1 | 3 | 9 |
| | ITGA8 | 0 | 0 | 0 | 0 |
| | ITGA9 | 0 | 0 | 0 | 1 |
| | ITGA10 | 0 | 0 | 2 | 2 |
| | ITGA11 | 0 | 0 | 0 | 0 |
| | ITGAD | 0 | 0 | 0 | 0 |
| | ITGAE | 22 | 28 | 20 | 18 |
| | ITGAL | 7 | 5 | 10 | 7 |
| | ITGAM | 0 | 0 | 0 | 6 |
| | ITGAV | 47 | 50 | 85 | 55 |
| | ITGAX | 0 | 0 | 0 | 0 |
| | ITGB1 | 328 | 481 | 389 | 378 |
| | ITGB1BP1 | 80 | 85 | 53 | 52 |
| | ITGB1BP2 | 0 | 0 | 1 | 0 |
| | ITGB1P1 | 0 | 0 | 0 | 1 |
| | ITGB2 | 6 | 12 | 20 | 20 |
| | ITGB3 | 0 | 0 | 0 | 0 |
| | ITGB3 | 0 | 0 | 0 | 0 |
| | ITGB3BP | 24 | 24 | 18 | 8 |
| | ITGB4 | 2 | 2 | 4 | 13 |
| | ITGB5 | 69 | 82 | 142 | 130 |
| | ITGB6 | 0 | 0 | 0 | 0 |
| | ITGB7 | 1 | 0 | 1 | 3 |
| | ITGB8 | 0 | 0 | 0 | 0 |
| | ITGBL1 | 0 | 0 | 0 | 0 |

| | Gene | FPKM Non_Sphe | Dx_Sphe | Non_Mono | Dx_Mono |
|---|---|---|---|---|---|
| Proteoglycan | CSPG4P4Y | 0 | 0 | 0 | 0 |
| | CSPG4 | 0 | 0 | 0 | 1 |
| | CSPG4P1Y | 0 | 0 | 0 | 0 |
| | CSPG4P2Y | 0 | 0 | 0 | 0 |
| | CSPG4P3Y | 0 | 0 | 0 | 0 |
| | CSPG4P5 | 0 | 0 | 0 | 0 |
| | CSPG5 | 0 | 1 | 0 | 5 |
| | HAPLN1 | 0 | 0 | 0 | 0 |
| | HAPLN2 | 0 | 0 | 0 | 3 |
| | HAPLN3 | 0 | 0 | 0 | 0 |
| | HAPLN4 | 0 | 0 | 0 | 0 |
| | HSPG2 | 30 | 54 | 148 | 320 |
| | IMPG1 | 0 | 0 | 0 | 0 |
| | IMPG2 | 0 | 0 | 0 | 0 |
| | IMPG2 | 0 | 0 | 0 | 0 |
| | LEPRE1 | 61 | 96 | 120 | 178 |
| | PRG2 | 0 | 0 | 0 | 0 |
| | PRG3 | 0 | 0 | 0 | 0 |
| | PRG4 | 0 | 0 | 0 | 0 |
| | SPOCK1 | 0 | 0 | 0 | 0 |
| | SPOCK2 | 34 | 55 | 192 | 311 |
| | SPOCK3 | 0 | 0 | 0 | 0 |
| | TSKU | 42 | 5 | 44 | 50 |

| | Gene | FPKM Non_Sphe | Dx_Sphe | Non_Mono | Dx_Mono |
|---|---|---|---|---|---|
| Collagen | COL1A1 | 0 | 0 | 1 | 3 |
| | COL1A2 | 0 | 0 | 0 | 1 |
| | COL2A1 | 171 | 312 | 157 | 288 |
| | COL4A1 | 0 | 0 | 0 | 0 |
| | COL4A2 | 1 | 0 | 1 | 1 |
| | COL4A3 | 0 | 0 | 0 | 0 |
| | COL4A4 | 0 | 0 | 0 | 0 |
| | COL4A5 | 1 | 1 | 0 | 1 |
| | COL4A6 | 0 | 0 | 0 | 0 |
| | COL5A1 | 0 | 1 | 1 | 1 |
| | COL5A2 | 1 | 1 | 1 | 2 |
| | COL5A3 | 0 | 0 | 0 | 0 |
| | COL6A1 | 3 | 5 | 5 | 23 |
| | COL6A2 | 0 | 0 | 0 | 2 |
| | COL6A5 | 0 | 0 | 0 | 0 |
| | COL6A6 | 0 | 0 | 0 | 0 |
| | COL7A1 | 15 | 21 | 73 | 97 |
| | COL8A1 | 0 | 0 | 0 | 0 |
| | COL8A2 | 0 | 0 | 0 | 1 |
| | COL9A1 | 0 | 0 | 0 | 1 |
| | COL9A3 | 84 | 141 | 293 | 437 |
| | COL9A2 | 12 | 16 | 45 | 76 |
| | COL10A1 | 0 | 0 | 0 | 0 |
| | COL11A1 | 0 | 0 | 0 | 0 |
| | COL11A2 | 0 | 0 | 1 | 3 |
| | COL12A1 | 0 | 0 | 0 | 0 |
| | COL13A1 | 0 | 0 | 0 | 0 |
| | COL14A1 | 0 | 0 | 0 | 1 |
| | COL15A1 | 0 | 0 | 0 | 0 |
| | COL16A1 | 7 | 8 | 7 | 11 |
| | COL17A1 | 0 | 0 | 0 | 1 |
| | COL18A1 | 55 | 100 | 175 | 200 |
| | COL19A1 | 0 | 0 | 0 | 0 |
| | COL20A1 | 0 | 0 | 0 | 3 |
| | COL21A1 | 78 | 116 | 97 | 68 |
| | COL22A1 | 0 | 0 | 0 | 0 |
| | COL23A1 | 0 | 0 | 0 | 0 |
| | COL24A1 | 0 | 0 | 0 | 0 |
| | COL25A1 | 0 | 0 | 0 | 0 |
| | COL27A1 | 55 | 57 | 144 | 248 |
| | COL28A1 | 0 | 0 | 0 | 1 |

Extended Data Figure 11. Systematic comparison of the transcription mass (FPKM) of the ECM-related gene series expressed in HepG2 cells under several culture conditions. All data were obtained from RNA-seq results.

# Extended Data Figure 12

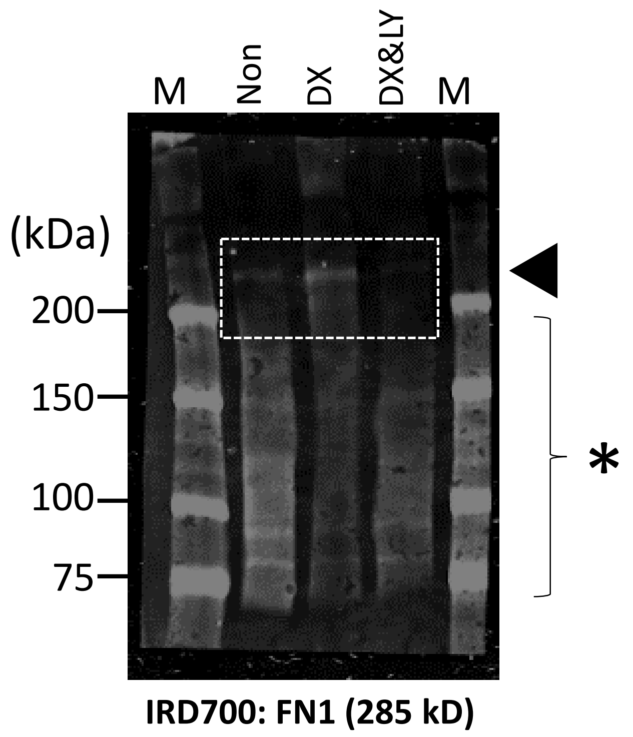


Extended Data Figure 12. The whole image of immunoblotting analysis of fibronectin expression shown in Fig. 4g. Arrowhead: non-disassembled FN (285 kDa). Asterisk: a putative disassembled FN. Dashed rectangle: area shown in Fig. 4g. The image was prepared by Odyssey system (LI-COR, Lincoln, NB, USA).

# Extended Data Figure 13

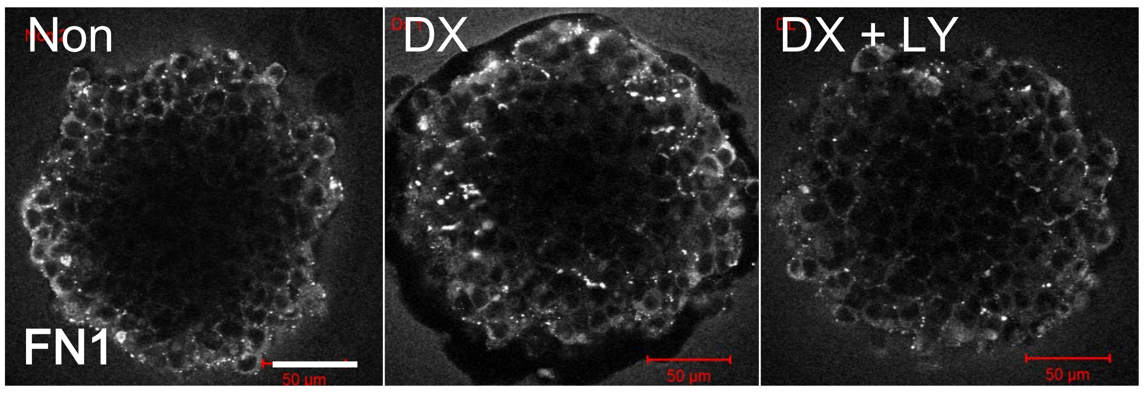


Extended Data Figure 13. Immunofluorescence of fibronectin in HepG2 spheroids after drug treatment. Day 5 HepG2 spheroids of non-treated or treated with DX or with DX and the TGFR inhibitor (LY) were embedded in the Smear Gell, fixed, permeabilized with detergent, and immunostained with rat anti-FN1 antibody and fluorescence-conjugated secondary antibody. Images were acquired using the confocal microscopy. Scale bar: 50 µm. .The images were prepared by ZEN software (Zeiss, Jena, Germany),
